# Asparagine and Glutamine Side-Chains and Ladders in HET-s(218–289) Amyloid Fibrils Studied by Fast Magic-Angle Spinning NMR

**DOI:** 10.3389/fmolb.2020.582033

**Published:** 2020-09-30

**Authors:** Thomas Wiegand, Alexander A. Malär, Riccardo Cadalbert, Matthias Ernst, Anja Böckmann, Beat H. Meier

**Affiliations:** ^1^Physical Chemistry, Eidgenössische Technische Hochschule (ETH) Zurich, Zurich, Switzerland; ^2^Molecular Microbiology and Structural Biochemistry, UMR 5086 CNRS/Université de Lyon, Labex Ecofect, Lyon, France

**Keywords:** solid-state NMR, fast MAS, amyloid fibrils, hydrogen bond, asparagine ladder

## Abstract

Asparagine and glutamine side-chains can form hydrogen-bonded ladders which contribute significantly to the stability of amyloid fibrils. We show, using the example of HET-s(218–289) fibrils, that the primary amide side-chain proton resonances can be detected in cross-polarization based solid-state NMR spectra at fast magic-angle spinning (MAS). *J*-coupling based experiments offer the possibility to distinguish them from backbone amide groups if the spin-echo lifetimes are long enough, which turned out to be the case for the glutamine side-chains, but not for the asparagine side-chains forming asparagine ladders. We explore the sensitivity of NMR observables to asparagine ladder formation. One of the two possible asparagine ladders in HET-s(218–289), the one comprising N226 and N262, is assigned by proton-detected 3D experiments at fast MAS and significant de-shielding of one of the NH_2_ proton resonances indicative of hydrogen-bond formation is observed. Small rotating-frame ^15^N relaxation-rate constants point to rigidified asparagine side-chains in this ladder. The proton resonances are homogeneously broadened which could indicate chemical exchange, but is presently not fully understood. The second asparagine ladder (N243 and N279) in contrast remains more flexible.

## Introduction

**FIGURE 1:**
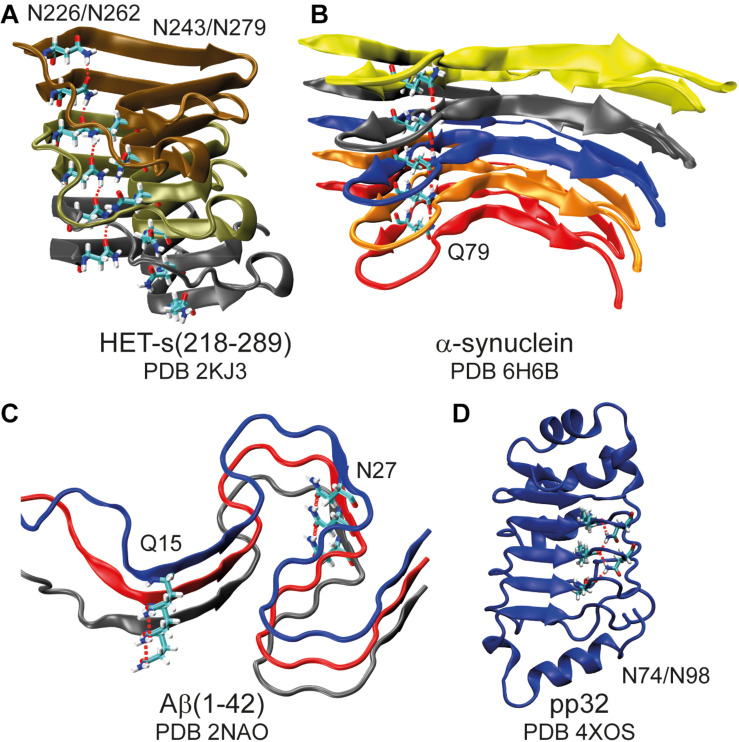
Asparagine and glutamine ladders in protein structures. Schematic representation of amyloid fibrils **(A–C)** highlighting asparagine and glutamine ladders and a leucine-rich repeat protein in which two asparagine side-chains constitute to a second backbone **(D)**. Hydrogen bonds are highlighted by red dashed lines. Note that for **(A)** structure 16 of the lowest energy structural bundle is shown as it particularly nicely illustrates the ladder motif (for more structures see Supplementary Figure S1).

The terminal primary amide group of asparagine and glutamine side-chains can function as both, hydrogen-bond donor via the NH_2_ group as well as acceptor via the carbonyl group and thus these side-chains are important for protein stability (Vernet et al., 1995; Worth and Blundell, 2010), protein-protein (Van Melckebeke et al., 2010; Talavera et al., 2011; Wälti et al., 2016), protein-ligand (Higman et al., 2004; Raymond et al., 2004) and protein RNA/DNA interactions (Luscombe et al., 2001; Lejeune et al., 2005). In amyloids they can form asparagine/glutamine ladders (Wan and Stubbs, 2014; Riek and Eisenberg, 2016; Eisenberg and Sawaya, 2017; Kurt et al., 2017), e.g., in α-synuclein (Guerrero-Ferreira et al., 2018, 2019), Aβ(1–42) (Colvin et al., 2016; Wälti et al., 2016), the functional amyloid HET-s(218–289) (Wasmer et al., 2008; Van Melckebeke et al., 2010), and the polyglutamine rich Orb2 (Hervas et al., 2020) (for some representative structures see Figure 1 and Supplementary Figure S1). Such side-chains play also important roles in many other amyloids, including the yeast prion Sup35 (Luckgei et al., 2013), huntingtin (Hoop et al., 2016), and prions (Kurt et al., 2017), where these residues form the fibril core, ladders and steric zippers (Sawaya et al., 2007).

Solid-state nuclear magnetic resonance (NMR) spectroscopy is a suitable technique to identify asparagine/glutamine ladders in amyloid fibrils: the ^1^H chemical-shifts are sensitive reporters and less-shielded ^1^H resonances indicate a participation of the proton in a hydrogen bond (Wagner et al., 1983) whereas relaxation properties can describe dynamical effects. Such spectroscopic information extends the structure information from NMR and cryo-EM (Gremer et al., 2017; Guerrero-Ferreira et al., 2018, 2019; Kollmer et al., 2019; Schmidt et al., 2019; Hervas et al., 2020). Infrared spectroscopy (IR) has also been used recently to identify glutamine ladders in amyloid-forming peptides (Wu et al., 2019) and solution-state NMR has been used to probe asparagine side-chain properties engaged in a dense hydrogen-bond network in the leucine-rich repeat (LRR) proteins pp32 (Klein et al., 2019) (see also Figure 1D). The detection of glutamine and asparagine side-chains by solution-state NMR spectroscopy, e.g., in ^15^N,^1^H HSQC-type experiments, has been employed in a variety of biological contexts to probe their structural and dynamic properties (Buck et al., 1995; Vance et al., 1997; Cai et al., 2001; Mulder et al., 2001; Higman et al., 2004; Klein et al., 2019). We show that fast magic-angle spinning (MAS) (Agarwal et al., 2014; Andreas et al., 2015, 2016; Böckmann et al., 2015; Sternberg et al., 2018; Stöppler et al., 2018; Malär et al., 2019; Penzel et al., 2019; Vasa et al., 2019) can detect asparagine and glutamine side-chains in cross-polarization (CP) solid-state NMR proton spectra and, sometimes, also in *J*-coupling based experiments, in particular INEPT (Morris and Freeman, 1979) experiments, which then can be employed to distinguish the two NH_2_ side-chain proton resonances from amide NH protons. In amyloid fibrils of HET-s(218–289) of the filamentous fungus *Podospora anserina*, the asparagine side-chains are visible in CP-based experiments, but not in INEPT experiments at an MAS frequency of 110 kHz. We discuss NMR spectral and relaxation parameters determined for asparagine side-chains forming asparagine ladders, such as high-frequency shifted proton resonances and small rotating-frame ^15^N relaxation-rate constants.

## Results and Discussion

### Assignment of Asparagine and Glutamine Residues in HET-s(218–289)

**FIGURE 2:**
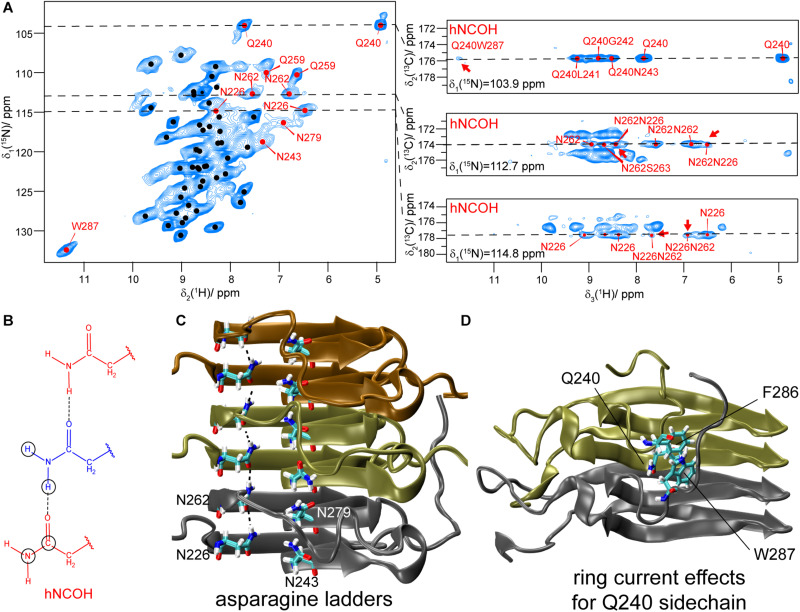
Asparagine and glutamine side-chains in HET-s(218–289) fibrils. **(A)** ^1^H,^15^N CP-hNH spectrum of HET-s fibrils (left) and 2D planes of 3D hNCOH spectra showing spatial proximities for N226, N262 and Q240 (right, the cross peaks mentioned in the text are highlighted by red arrows). The spectra were recorded at a MAS frequency of 110 kHz and an external magnetic field of 20.0 T. The black dots indicate assigned backbone amide correlations based on the BMRB accession code 26913 (Smith et al., 2017). **(B)** Schematic drawing of an asparagine ladder. Circles indicate connectivities as expected for 3D hNCOH experiments. **(C)** Asparagine ladders in HET-s highlighted on the PDB structure (PDB accession code 2KJ3). **(D)** Orientation of the Q240 side-chain toward aromatic residues in HET-s(218–289) highlighted on the PDB structure.

The side-chain amide protons of asparagine (N) and glutamine (Q) side-chains can be observed in proton-detected fast MAS experiments. CP hNH correlation spectra of deuterated and 100% back-exchanged, as well as uniformly ^13^C and ^15^N labeled HET-s(218–289) fibrils (abbreviated in the following with DUL), recorded at a MAS frequency of 110 kHz, are shown in Figure 2A. N/Q side-chains resonate in a region somewhat more shielded than backbone amides for both ^15^N and ^1^H shifts although the regions overlap. Some of the observed N/Q side-chains (e.g., Q240, N226, N262) lead to two proton correlation peaks with a common ^15^N frequency indicating no or only a slow rotation around the C^γ^ -N^δ2^ (N) or C^δ^ -N^ε2^ (Q) bond as shown in solution-state experiments (Guenneugues et al., 1997). The side-chain assignment is based on a NCOCX 3D experiment performed on a fully protonated HET-s(218–289) sample shown in Supplementary Figure S2 which yields the ^15^N side-chain resonances. The proton chemical-shift values are extracted from the 2D hNH spectrum (Figure 2A). In total, four out of five asparagine side-chains (N226, N243, N262, N279) and the two glutamine side-chains (Q240, Q259) were identified in the hNH spectrum, only the very C-terminal N289 remains absent probably due to large dynamics (Van Melckebeke et al., 2010). The side-chains of N243, Q259 and N279 have however lower intensity (Supplementary Figure S3 for signal-to-noise ratios extracted from 2D spectra) and remain undetected in proton-detected 3D spectra and thus only one NH_2_ proton is assigned for N243 and N279. Note that the second NH_2_ resonance of Q259 is not visible in the CP spectrum, but can be identified in an INEPT-based hNH spectrum (*vide infra*).

**FIGURE 3:**
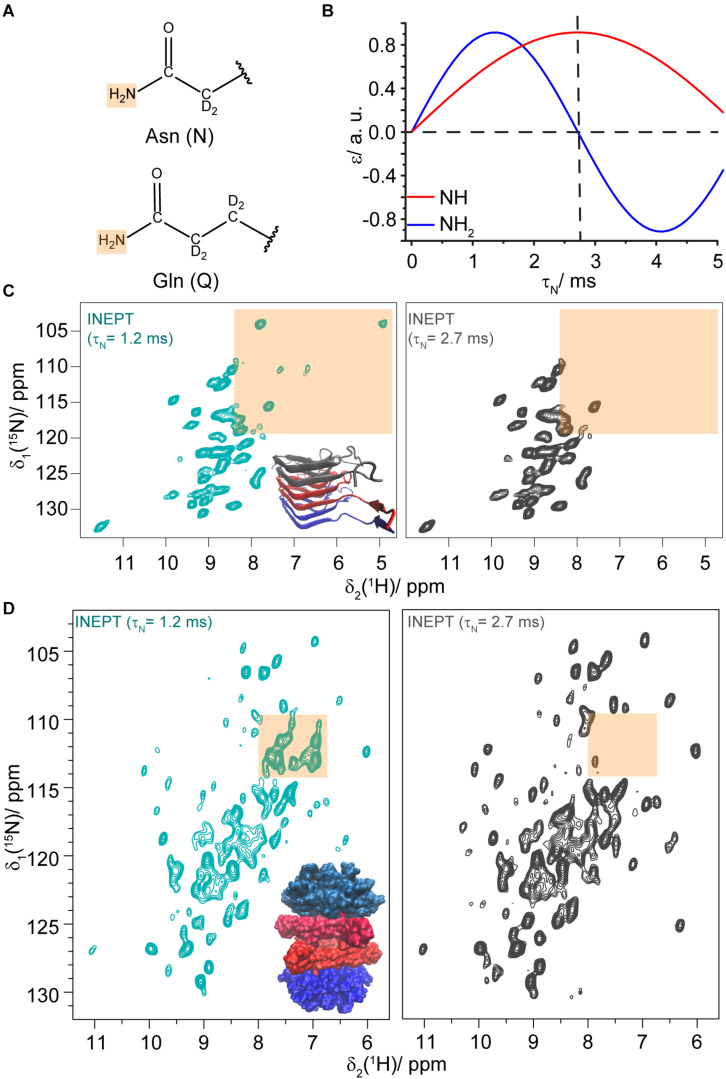
Asparagine and glutamine side-chains identified in INEPT experiments. **(A)** Chemical structures of asparagine and glutamine side-chains. **(B)** Dependence of the INEPT enhancement ε on the τ_N_ mixing time for NH (blue) and NH_2_ (red) spin pairs. τ_H_ was set to 2 ms, a ^15^N-^1^H *J* coupling constant of 92 Hz was assumed and relaxation effects were neglected. **(C)** ^15^N,^1^H INEPT-hNH spectra of DUL HET-s(218–289) fibrils recorded with different τ_N_ mixing times (τ_H_ was set to 2 ms). The N/Q side-chain region is highlighted in orange. **(D)** ^15^N,^1^H INEPT-hNH spectra of DUL DnaB with different τ_N_ mixing times. The N/Q spectral region is highlighted in orange. The spectra were recorded at a MAS frequency of 110 kHz and an external magnetic field of 20.0 T.

We next address the question whether *J*-coupling based INEPT-hNH experiments are also suitable to detect N/Q side-chains, similar to ^15^N,^1^H HSQC experiments applied in solution-state NMR. The well-known differences in the magnetization-transfer curves of NH and NH_2_ side-chain resonances in a refocused INEPT-based hNH experiment (Sørensen and Ernst, 1983) could then be used to distinguish the NH_2_ proton resonances of N/Q side-chains from spectrally overlapping backbone NH resonances (for the chemical structures of N/Q side-chains see Figure 3A). By setting the INEPT delay *τ*_N_ (Supplementary Figure S4) to 2.7 ms ($\frac{1}{4J_{\text{NH}}}$), the NH_2_ resonances will be strongly suppressed in the INEPT transfer; in contrast, when setting *τ*_N_ close to 1.4 ms, they appear in the spectra (see Figure 3B). For HET-s(218–289) only two of the seven NH_2_ pairs (assigned to Q240 and Q259) are visible in INEPT experiments (see Figure 3C). None out of the five asparagine residues is detected indicating that proton transverse magnetization lifetimes are too short to detect the HET-s(218–289) asparagine side-chains in INEPT. We attribute this to dynamics as discussed below. Sample deuteration as used herein is crucial for INEPT experiments to prolong proton transverse magnetization lifetimes.

We envision that such INEPT-filtering experiments might also be helpful for large proteins to identify and further characterize the corresponding N/Q side-chain region in crowded NMR spectra. To test the feasibility of such an approach, we recorded INEPT-hNH spectra for the hexameric 354 kDa protein DnaB (Wiegand et al., 2019; Wiegand, 2020) (complexed with ADP:AlF_x_^–^ and DNA). Figure 3D shows a comparison of the spectra with *τ*_N_ = 1.2 ms (cyan) and *τ*_N_ = 2.7 ms (gray, for a difference spectrum see Supplementary Figure S5 and for further *τ*_N_-times see Supplementary Figures S6, S7) and indeed several N/Q side-chains are identified at 110 kHz MAS. We note that the INEPT spectra of this 488 residue protein (with 24 N and 23 Q residues) are again rather sparse, also for the backbone amides, as the INEPT step filters out resonances with short transverse proton magnetization lifetimes that are on the other hand visible in CP-hNH spectra (Wiegand et al., 2020).

### Asparagine Ladders

**FIGURE 4:**
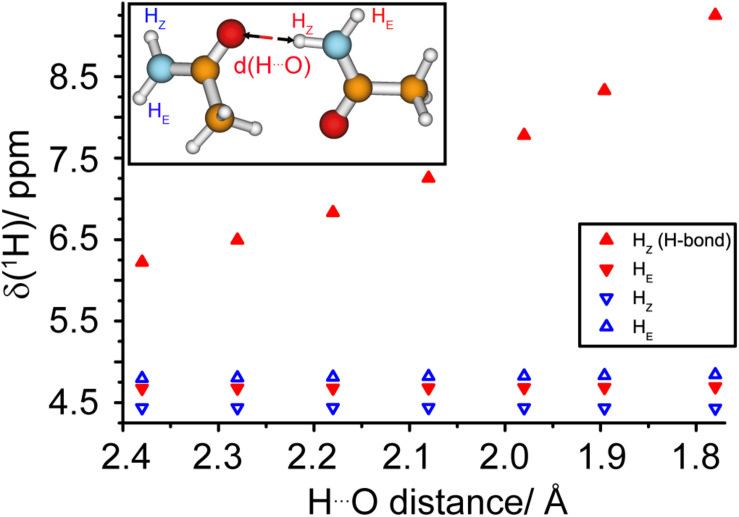
The proton chemical shift is a sensitive tool to measure hydrogen bond strengths in asparagine ladders. DFT calculations (B3-LYP/def2-TZVP) of ^1^H chemical-shift values as a function of the hydrogen bond length in a model representing two asparagine side-chains. The geometries of the two molecules were fixed and the two fragments were moved relative to each other along the N-H^…^ O distance vector, see insert.

In the amyloid fibrils of HET-s(218–289), two asparagine ladders (one comprising N226 and N262 and the other N243 and N279) have been postulated from the structure obtained by solid-state NMR. N226 and N262 point to the inside of the fibril and are located next to the hydrophobic core, whereas N243 and N279 are outside of the core (see Figure 2C; Wasmer et al., 2008). The NMR structure shows a well-formed ladder for N226/N262 in most structures of the structural bundle whereas N243/N279 do not form a regular ladder. It is, at this point not *a priori* clear if this difference is a consequence of the limited precision of the NMR structure or if it reflects real structural differences. Such small, but potentially important structural features are not easily addressed by cryo-EM or NMR structures. However, spectroscopic features may be exploited: the proton chemical shift itself is highly sensitive to changes in the environment and can be used as an indicator for hydrogen bonding (Cordier and Grzesiek, 1999) characteristic for ladders (see Figure 2). As illustrated in Density Functional Theory (DFT) calculations on a model consisting of two asparagine side-chains engaged in a hydrogen bond, see Figure 4 and Supplementary Figure S8, a significant de-shielding of the H_Z_ proton (see insert of Figure 4 for the nomenclature) participating in a hydrogen bond is observed. In one calculation, two asparagine side-chain fragments were moved relative to each other along the N-H^…^O distance vector (Figure 4), while in the second calculation only the H_Z_ proton was moved along that vector and all other atoms of the two fragments were kept fixed (Supplementary Figure S8). Both cases reveal that the shorter the hydrogen bond, the larger the chemical-shift difference between the H_Z_ and H_E_ protons (Barfield, 2002). We speculate that the scenario described in Figure 4 is more likely reflecting the situation in asparagine ladders, although not only the hydrogen bond length effects the strength of the hydrogen bond and thus the proton chemical-shift values observed experimentally, but also the bond angle certainly has an influence.

Experimentally, for N226 we find a large difference between the two proton resonances resonating at 8.4 and 6.5 ppm. For N262 the less-shielded value is 7.6 ppm compared to 6.8 ppm for the more-shielded one. The two NH_2_ protons are thus shifted in different directions with respect to their average chemical-shift values over all deposited asparagine residues as extracted from the BMRB database (7.3 and 7.1 ppm)^1^.

3D hNCOH experiments (Figure 2B) do not only identify the two protons in the same NH_2_ group, but also probe the spatial proximity between such side-chains. A 9 ms ^13^CO-^1^H CP-step was employed to achieve a polarization transfer to both NH_2_ protons of the neighboring asparagine side-chain within the same ladder. Similar experiments have been described recently to identify backbone hydrogen bonding patterns in SH3 and GB1 (Friedrich et al., 2020). And indeed, the ^15^N planes at the NH_2_ side-chain chemical-shift value of N262 (112.7 ppm) and N226 (114.8 ppm) clearly show cross peaks to the proton shifts of the NH_2_ group of N226 and N262, respectively (see Figure 2A, red arrows) indicating their spatial proximity. In HET-s(218–289) this information corroborates the known register in the β-sheets and in other systems of unknown structure this may be of even more interest. For residues N243/N279 that form the second asparagine ladder no signals are detected in the CP-based 3D hNCOH spectrum.

### Homogeneous Proton Linewidths of Asparagine and Glutamine Side-Chains

The visibility of the proton resonances in INEPT-type experiments is related to the homogeneous proton linewidth (Δ^homo^) which is determined by the geometry of the proton dipolar network (coherent contribution; Zorin et al., 2006; Sternberg et al., 2018; Malär et al., 2019), additional additive contributions resulting from Redfield-type relaxation caused by stochastic molecular motion (incoherent contribution from anisotropic interactions) and chemical-exchange broadening effects (incoherent contributions from isotropic chemical-shift differences) (Sternberg et al., 2018; Malär et al., 2019; Penzel et al., 2019). Unfortunately, the three contributions cannot easily be addressed separately, and only their sum is observed experimentally. Coherent and incoherent contributions are expected to decrease with faster spinning (Lewandowski et al., 2011; Andreas et al., 2015; Böckmann et al., 2015; Sternberg et al., 2018; Malär et al., 2019; Penzel et al., 2019) while chemical exchange is largely MAS-frequency independent. Spin-echo decay experiments allow determination of proton *T*_2_′ relaxation times and Δ^homo^ = *R*_2_′/π = 1/(π*T*_2_′) values. The pulse sequences used to measure Δ^homo^ values are shown in Supplementary Figure S9.

**FIGURE 5:**
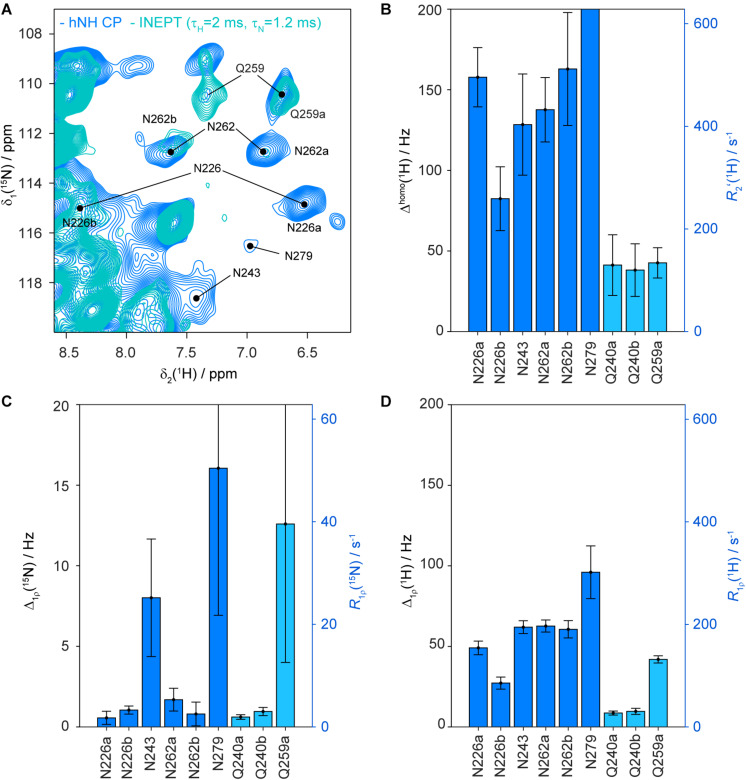
Homogeneous proton linewidths and ^15^N and ^1^H R_1ρ_ rate constants of asparagine and glutamine side-chains in HET-s(218–289) fibrils. Zoom into INEPT-hNH (cyan) and CP-hNH (blue) spectra of HET-s(218–289) fibrils **(A)** showing the asparagine and glutamine side-chain region and some resonance assignments. Homogeneous proton linewidths for asparagine and glutamine side-chains of HET-s(218–289) fibrils are given in **(B)**. Nitrogen spin-lock relaxation rate constants of asparagine and glutamine side-chains are shown in **(C)**, proton spin-lock relaxation times in **(D)**. All error bars shown correspond to two standard deviations. The relaxation rate constants given on the vertical axis are related according to Δ = *R*/π. The relaxation times were determined at a MAS frequency of 105 kHz and an external magnetic field of 20.0 T.

For HET-s(218–289) fibrils, Δ^homo^ values between 80 and 160 Hz for the N226, N243 and N262 side-chain protons are observed (see Figures 5A,B) which explains why these resonances remain absent in INEPT experiments (a Δ^homo^ of 160 Hz, *T*_2_′∼2 ms, would lead to an attenuation of ∼88% in the refocused INEPT efficiency with INEPT delays of τ_H_ = 2.0 ms and τ_N_ = 1.2 ms). Δ^homo^ for N279 is even larger (543 ± 140 Hz). All these values are significantly larger than the corresponding values of the backbone amide protons, which have been reported before, showing on average 22 ± 1 Hz at 100 kHz MAS for DUL HET-s(218–289) (Smith-Penzel, 2019). The glutamine side-chains which are easily observed in INEPT-hNH have, as expected, a smaller Δ^homo^ value (<50 Hz). As an additional reference we also measured the homogeneous proton linewidths for asparagine and glutamine side-chains in DnaB. In accordance with the experimental observation of glutamine side-chains in INEPT spectra, the Δ^homo^ are indeed smaller and amount on average to 31 ± 5 Hz (Supplementary Figure S10).

To investigate whether the larger-than-expected Δ^homo^ values observed for the asparagine side-chains are related to coherent broadening mechanisms, we have numerically simulated the ^1^H solid-state NMR spectrum using the geometry of the six NH_2_ proton spins of N262-N226-N262 taken from the PDB file (Figure 1, PDB accession number 2KJ3, the chosen geometry is taken from structure 16 in which the asparagine ladder N226/N262 is most clearly visible, see also Supplementary Figure S11A). Those simulations yield Δ^homo^ values of less than 5 Hz (see Supplementary Figure S11B) which illustrates that the main contribution to Δ^homo^ is not related to coherent effects resulting from the geometry within an asparagine ladder even if the contribution from a close-by backbone amide is taken into account (Supplementary Figure S12). The numerically simulated spin-echo decay profile using the same six-spin system are shown in Supplementary Figure S11C and also give a negligible Δ^homo^ value of 2 Hz. Cross terms between heteronuclear ^15^N-^1^H dipolar and/or *J*-couplings and the proton homonuclear dipolar couplings were also not found to make an appreciable contribution (Supplementary Figure S11C).

### ^15^N Rotating-Frame Relaxation Rates of Asparagine and Glutamine Side-Chains: Rigid Side-Chains in Asparagine Ladders

^15^N rotating-frame relaxation rate constants, characterized by *R*_1ρ_, may give further information about the dynamics of asparagine and glutamine side-chains (for the pulse sequences see Supplementary Figure S13 and for the site-specific decay curves Supplementary Figures S14, S15). Under the experimental conditions used (110 kHz MAS, 13 kHz rf-field amplitude for the spin lock) the experiment is sensitive to dynamics with correlation times in the hundreds of nanoseconds to low microsecond range (Lakomek et al., 2017). Figure 5C shows the ^15^N *R*_1ρ_ experimental relaxation-rate constants for HET-s(218–289) fibrils. Typical rigid backbone nitrogen atoms possess *R*_1ρ_ values of around 2 s^−1^ (Schanda and Ernst, 2016; Lakomek et al., 2017). The values for the side-chain NH_2_ nitrogen of N226 and N262 are also in the order of 2 s^–1^ indicating the absence of dynamics on this timescale for side-chain nitrogen (for details on relaxation properties of NH_2_ spin systems see Bull (1992). Interestingly, N243 and N279 show significantly larger ^15^N *R*_1ρ_ (>25 s^–1^) pointing to a higher degree of flexibility of these side-chains. Based on the measured *R*_1ρ_ values, the side-chain of Q240 is similarly rigid as N226 and N262, whereas Q259 is more flexible and shows weaker correlations in ^13^C-detected 3D spectra (see Supplementary Figure S2). In DnaB, which does not contain N/Q ladders, the side-chain nitrogen *R*_1ρ_ relaxation-rate constants are ranging between 10 and 32 s^–1^ (see Supplementary Figure S10C) and are, thus, larger than for the rather rigid N226/N262 side-chains of HET-s(218–289) fibrils.

The higher rigidity of the ladder N226/N262 compared to N243/N279 might be a consequence of a higher water accessibility of N243/N279 in agreement with their location outside of the hydrophobic core. All asparagine residues influence the fibril stability in the presence of guanidine as a chemical denaturant as shown by site-directed mutagenisis (Wan and Stubbs, 2014). However, mutation of N226 and N262 yields more destabilization than mutation of N243 and especially N279, in the latter mutant a similar chemical stability than in wild-type HET-s(218–289) fibrils was observed (Wan and Stubbs, 2014).

Since so far coherent and incoherent broadening mechanisms cannot account for the observed proton homogeneous linewidth, we also consider the possibility of chemical-exchange broadening, namely slow to intermediate exchange (below coalescence) on a millisecond timescale to explain the broader-than-expected lines. Such processes have been observed for asparagine side-chains in T4 lysozyme (Mulder et al., 2001) or in coiled-coil homo dimers (Thomas et al., 2017) in solution. The exchange is most likely asymmetric with one dominating conformer which is detected in our spectra. In our model, the conformational states represent asparagine side-chains hydrogen-bonded in a ladder (the most populated state observed experimentally) and an additional so far unobserved state. We performed ^15^N chemical-exchange saturation transfer (CEST) experiments (Vallurupalli et al., 2012), but were not able to observe this state requiring further experimental work. The NH_2_ proton relaxation-rate constants in the rotating frame measured for the asparagine side-chains are larger compared to backbone amides possibly as a consequence of a modulation of the proton homonuclear dipolar couplings by the exchange process (see Figure 5D and Supplementary Figure S16). However, the exchange contribution to the measured proton *R*_1ρ_ values is negligible at a spin-lock field of 13 kHz as used in our experiment (Palmer and Massi, 2006). Whether this observation is a general feature of N/Q ladders has to be investigated on further amyloid fibrils in future work, it is however interesting that the glutamine side-chains in HET-s(218–289) do not show these effects, and their homogeneous linewidths are roughly by a factor of three smaller.

### Characterization of Glutamine Side-Chains and the Orientation of Q240

For the two glutamine side-chains of HET-s(218–289), no ladder formation is possible from the structure (Van Melckebeke et al., 2010). Both glutamine side-chain NH_2_ are observed in the CP and INEPT-spectra (Figures 2A, 3D). For Q240, one proton is shifted to the more shielded region (4.8 ppm), the other one is at 7.8 ppm, slightly less shielded compared to the average chemical shift values taken from the BMRB database (7.22/7.04 ppm). The increased shielding of one of the NH_2_ proton of Q240 might be associated with ring-current effects, since Q240 points toward a hydrophobic pocket constituted by aromatic residues (F286 and W287, Figure 2D; Van Melckebeke et al., 2010). The cross signal between Q240 and W287 in the 3D hNCOH experiment reveals that the side-chain of Q240 is indeed in spatial proximity to the side-chain NH group of W287 (Figure 2A).

## Conclusion

We detected asparagine and glutamine NH_2_ side-chains proton signals in fast MAS spectra of HET-s(218–289) in its amyloid form. The spectral and relaxation properties are described and the influence of the formation of ladders is investigated. Table 1 summarizes the NMR properties of such side-chains. The following observations were made for the asparagines involved in a well-formed ladder: (i) strong de-shielding of the proton shift of one of the NH_2_ protons, (ii) a large chemical-shift difference between the two protons, (iii) correlations in 3D through-space hCONH experiments, and (iv) small ^15^N *R*_1ρ_ relaxation-rate constants. These properties characterize the N226-N262 asparagine ladder. In addition (v) a large homogeneous proton linewidth and (vi) a large ^1^H *R*_1ρ_ relaxation-rate constants were found. ^15^N rotating-frame relaxation rate constants indicate that the side-chains involved into the N226-N262 asparagine ladder are more rigid than for the N243-N279 ladder which appear more dynamic not forming a strong restraining hydrogen network. The large homogeneous proton linewidths for asparagine side-chains involved in the ladder, not fully understood yet, could be related to millisecond timescale chemical exchange characteristic for protons engaged in an asparagine ladder in HET-s(218–289) fibrils. The glutamine side-chain amide protons of HET-s(218–289) not participating in such interactions are less homogeneously broadened and have linewidths comparable to the backbone NH protons. Fast MAS NMR experiments thus probe protein side-chain hydrogen bonding, in particular in the context of asparagine/glutamine ladder formation. While more systems must be investigated for a full understanding, the results on HET-s(218–289) clearly show the potential of the approach.

**TABLE 1:** Overview of asparagine and glutamine side-chain spectral and relaxation properties.

	N226	N262	N243	N279	Q240	Q259
Visible in CP-hNH?	Yes	Yes	Yes	Yes	Yes	Yes
Visible in INEPT-hNH?	No	No	No	No	Yes	Yes
δ(^15^N^δ2^/^15^N^ε2^)/ppm	114.9 (115.4^a^)	112.8 (113.5^a^)	118.9 (n.d.^a^)	116.5 (115.4^a^)	104.0	110.2 (110.8^a^)
δ(^13^C^γ^/^13^C^δ^)/ppm	177.6 (177.4^a^)	173.9 (176.5^a^)	176.1 (176.1^a^)	176.4 (176.3^a^)	175.5 (175.7^a^)	180.3 (180.3^a^)
δ(^1^H^δ21/δ22^/^1^H^ε21/ε22^)/ppm	8.4/6.5	7.6/6.8	7.4/n.d.	7.0 (broad)	7.8/4.9	7.3/6.7
Δ[δ(^1^H)]/ppm	1.9	0.8	n.d.	n.d.	2.9	0.6
Δ^homo^ (^1^H)/Hz^c^	157 ± 18	138 ± 20	128 ± 50	543 ± 140	41 ± 20	43 ± 10
	82 ± 18	160 ± 40			38 ± 18	
*R*_1ρ_ (^15^N)/s^–1^	2.6 ± 0.7^b^	3.8 ± 1.6^b^	25 ± 10	50 ± 30	2.5 ± 0.5^b^	40 ± 30
*R*_1ρ_ (^1^H)/s^–1^	120 ± 9^b^	196 ± 9^b^	190 ± 17	302 ± 52	29 ± 4^b^	132 ± 7

*^a^Determined on protonated HET-s fibrils in ^13^C-detected MAS experiments (BMRB accession code 11064). Differences to the values derived from ^1^H-detected experiments are related to temperature differences in the experiments. ^b^The average value over the relaxation rate constants for both resonances is given. The error is derived by error propagation of the individual experimental errors (a 2σ error is given). ^c^Δ^homo^ is calculated according to R_2_’/π = 1/(πT_2_’).*

## Materials and Methods

### Protein Preparations

The preparations of the DUL *Hp*DnaB:ADP:AlF_4_^–^:DNA (Wiegand et al., 2019) and DUL HET-s(218–289) (Wasmer et al., 2008) samples were prepared as described previously. In short, the proteins were recombinantly expressed in D_2_O in presence of deuterated ^13^C-glucose (2 g/L) and ^15^N-ammonium chloride (2 g/L) as sole sources of carbon-13 and nitrogen-15. In case of HET-s(218–289), fibrillization was performed in protonated solvent (50 mM citric acid at pH 5 in H_2_O) for 24 h which yields 100% back-exchanged fibrils (Smith et al., 2017). For DnaB, back-exchange was achieved by purifying the protein in a protonated buffer (2.5 mM sodium phosphate, pH 7.5, 130 mM NaCl). The complex formation with ADP:AlF_x_^−^ and DNA (a single-stranded DNA molecule with 20 thymine bases was used, purchased from Microsynth) is described in reference Wiegand et al. (2019). UL HET-s(218–289) fibrils were prepared identically, but only using H_2_O instead of D_2_O and protonated ^13^C-glucose (2 g/L) and ^15^N-ammonium chloride (2 g/L).

### Solid-State NMR

Solid-state NMR spectra were acquired at 20.0 T static magnetic-field strength using a Bruker 0.7 mm probe. The MAS frequency was set to 110 kHz. The 2D spectra were processed with the software TOPSPIN (version 3.5, Bruker Biospin) with a shifted (3.0) squared cosine apodization function and automated baseline correction in the indirect and direct dimensions. The sample temperature was set to 293 K (Böckmann et al., 2009). All spectra were analyzed with the software CcpNmr (Fogh et al., 2002; Vranken et al., 2005; Stevens et al., 2011) and referenced to 4,4-dimethyl-4-silapentane-1-sulfonic acid (DSS). Site-specific relaxation-rate constants were extracted from the 2D hNH relaxation experiments, using the INFOS software (Smith, 2017) (see Supplementary Figures S14, S15 for the mono-exponential fits of all relaxation decay curves for DUL HET-s). Error bars for all relaxation data have been derived by bootstrapping methods, using 500 iterations and are shown as 2σ, where σ denotes the obtained error. For more details see Supplementary Table S1. The NMR spectra can be accessed at https://doi.org/10.3929/ethz-b-000430151.

### Numerical Simulations

The numerical simulations of ^1^H MAS spectra were performed with SIMPSON (Bak et al., 2000) using six proton spins in the geometry extracted from the pdb file 2JK3 (structure 16). The spin-echo decay curves were obtained using the same proton six-spin system with and without three nitrogen spins. The simulations were performed in the GAMMA spin simulations environment (Smith et al., 1994). The program code for the simulations is provided in the Supplementary Material.

### DFT Calculations

The DFT calculations were performed with TURBOMOLE (Ahlrichs et al., 1989) version 6.4.0. Geometry optimizations were performed on a B3-LYP (Stephens et al., 1994)/def2-TZVP (Weigend and Ahlrichs, 2005) level employing the D3 dispersion correction (Grimme et al., 2010). In all TURBOMOLE SCF calculations, an energy convergence criterion of 10^–7^ E_h_ was used, and in the geometry optimizations, an energy convergence criterion of 10^–6^ E_h_ was employed. The integration grid was set to m4. Magnetic shieldings were calculated within the GIAO framework on the same theoretical level and converted into chemical-shift values according to δ = σ_ref_-σ with σ_ref_ being the magnetic shielding of the protons of TMS calculated at the same level of theory (σ_ref_ = 31.9 ppm). The orientation-dependence of the ^1^H chemical-shift values was studied by moving the two molecules relative to each other along the N-H^…^ O distance vector (Figure 4) or moving the proton only along that vector (Supplementary Figure S8). No additional geometry optimization was performed.

## Data Availability Statement

All datasets generated for this study are included in the article/Supplementary Material.

## Author Contributions

RC prepared the samples. TW and AM performed the NMR experiments. TW, AM, ME, AB, and BM analyzed the data. TW and ME performed the simulations. TW, AB, ME, and BM designed and supervised the research. All authors contributed to the writing of the manuscript.

## Conflict of Interest

The authors declare that the research was conducted in the absence of any commercial or financial relationships that could be construed as a potential conflict of interest.

## Footnotes

1 http://www.bmrb.wisc.edu
