# Supplementary material for: Asparagine and Glutamine Side-Chains and Ladders in HET-s(218–289) Amyloid Fibrils Studied by Fast Magic-Angle Spinning NMR
Source: Front Mol Biosci. 2020 Sep 30;7:582033. doi: 10.3389/fmolb.2020.582033 (PMC7556116; doi:10.3389/fmolb.2020.582033)
Supplement: Supplementary file 1 [file Data_Sheet_1.PDF]

Supplementary Materials Section

Asparagine and glutamine side-chains and ladders in HET-s(218-289) amyloid fibrils studied by fast magic-angle spinning NMR

Thomas Wiegand[a,*,#], Alexander A. Malär[a,#], Riccardo Cadalbert[a], Matthias Ernst[a], Anja Böckmann[b], and Beat H. Meier[a,*]

[a] *Physical Chemistry, ETH Zurich, 8093 Zurich, Switzerland*

[b] *Molecular Microbiology and Structural Biochemistry UMR 5086 CNRS/Université de Lyon, Labex Ecofect, 69367 Lyon, France*

[#] Equal contributions

[*]Corresponding authors: thomas.wiegand@phys.chem.ethz.ch, beme@ethz.ch

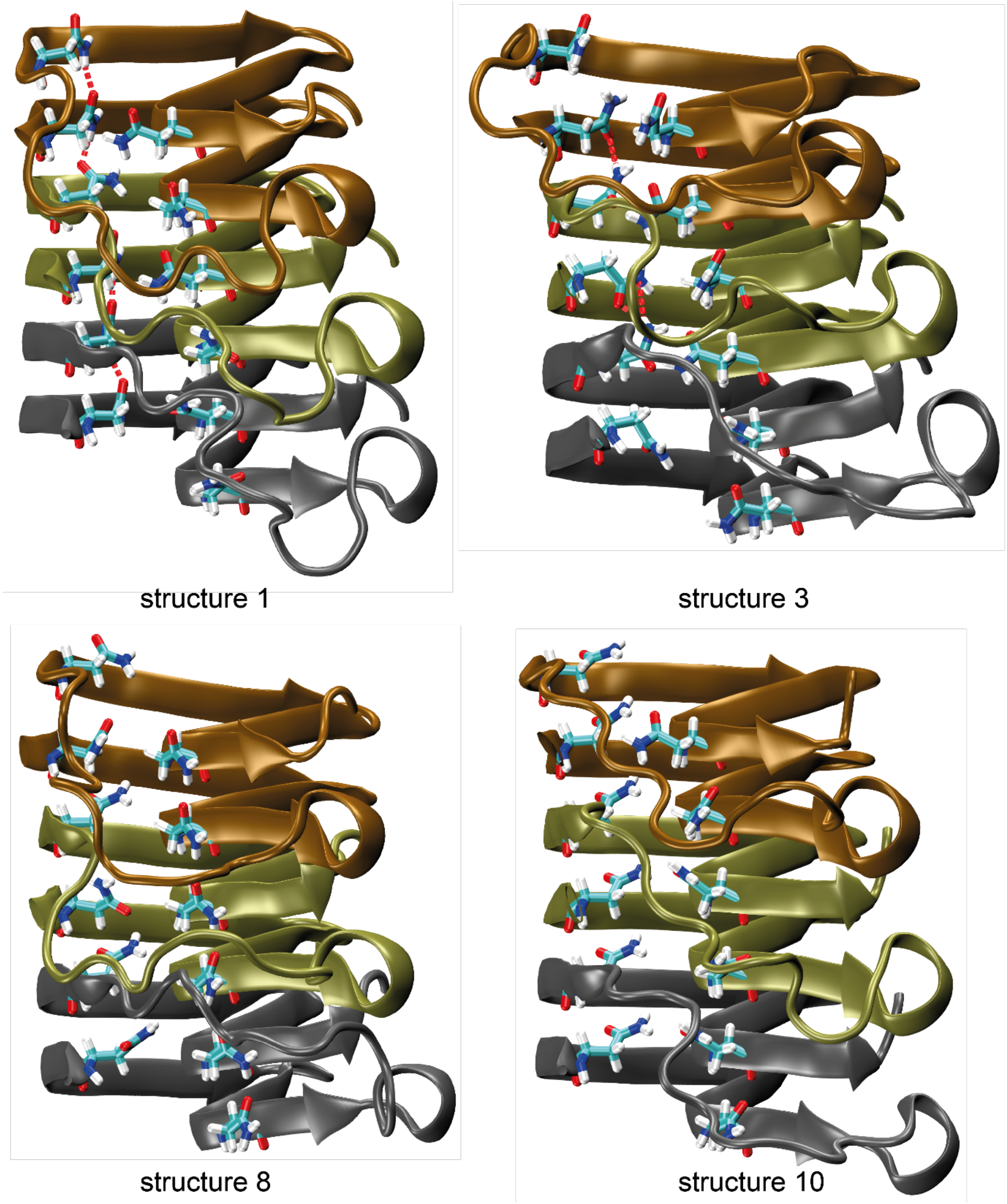

Figure S1: *Asparagine side-chain orientations differ in the structures derived from NMR.* Schematic representation of four representative structures of the structural bundle obtained by the NMR-based structure calculation of HET-s(218-289) fibrils (PDB accession number 2KJ3).

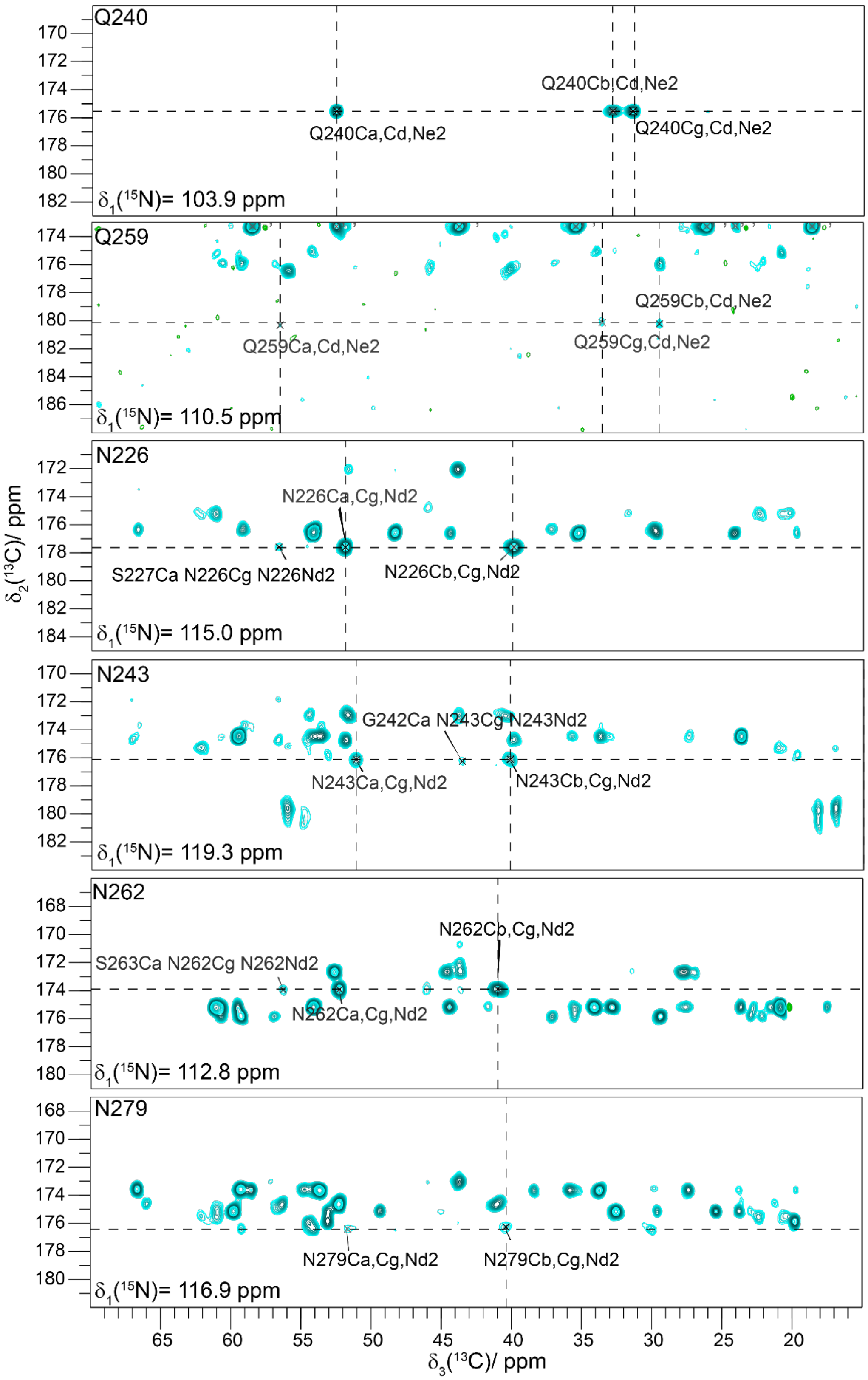

Figure S2: *Assignment of asparagine and glutamine side-chains in HET-s(218-289) fibrils by carbon-detected spectra.* 2D planes of a 3D carbon-detected NCOCX spectrum recorded at 17 kHz (employing an 80 ms Dipolar Assisted Rotary Resonance[1,2] mixing time) correlating side-chain $N^{\delta 2}$ and C^{γ} (N) or $N^{\varepsilon 2}$ and C^{δ} (Q) atoms with further atoms of the spin system. Such side-chains were only partially assigned in previous ^{13}C-detected experiments (BMRB accession code 11064)[3].

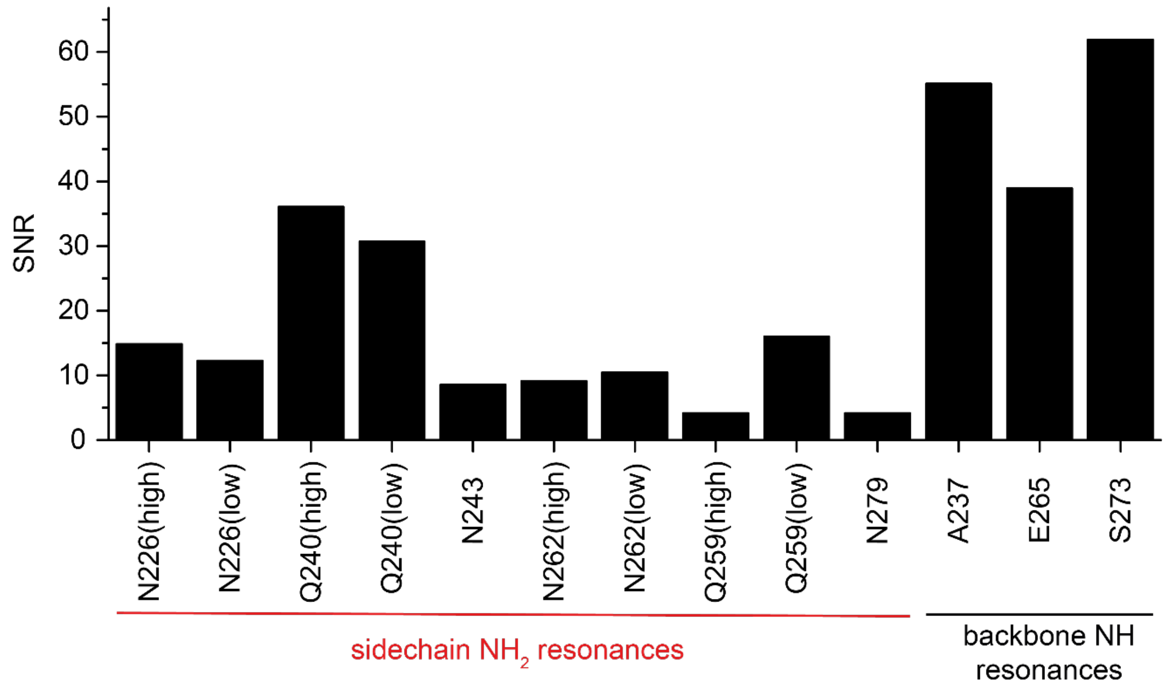

Figure S3: *Signal-to-noise ratios of NH_2 side-chain resonances of HET-s(218-289) fibrils in 2D hNH-CP experiments.* As a reference, the SNR-values for three backbone amides are shown. The values were extracted using CcpNmr.

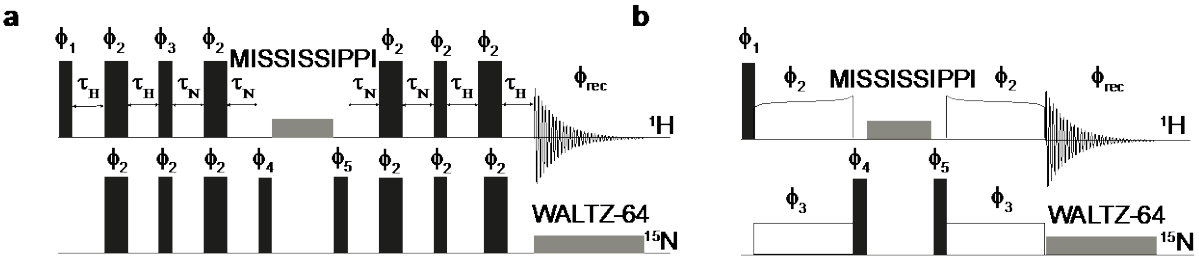

Figure S4: *INEPT and CP hNH pulse sequences.* The refocused INEPT pulse sequence is schematically shown in **a** and the CP pulse sequence in **b**. The phase parameters for **a** are given by $\phi_1 = x\,x - x - x$, $\phi_2 = x$, $\phi_3 = y$, $\phi_4 = y$, $\phi_5 = y - y$, $\phi_{\mathrm{rec}} = x - x\ - x\,x$ and in **b** by $\phi_1 = x\,x - x - x\,y\,y - y - y$, $\phi_2 = y\,y\,y\,y - x - x - x - x$, $\phi_3 = xx - x - x\,y\,y - y - y$, $\phi_4 = -y - y\,y\,y\,x\,x - x - x$, $\phi_5 = -y - y\,y\,y\,x\,x - x - x$, $\phi_{\mathrm{rec}} = x - x - x\,x\,y - y - y\,y$.

Note that in both cases 1D versions of the pulse sequences are shown. The transfer efficiency in refocused INEPT experiments is given for an NH spin pair by[4]

$$\varepsilon = \sin(2\pi J_{NH}\tau_H)\sin(2\pi J_{NH}\tau_N)\exp\left(\frac{-2\tau_H}{T_2'(H)}\right)\exp\left(\frac{-2\tau_N}{T_2'(N)}\right) \qquad (1)$$

and for NH_2 spin pairs by[4,5]

$$\varepsilon = 2\sin(2\pi J_{NH}\tau_H)\sin(2\pi J_{NH}\tau_N)\cos(2\pi J_{NH}\tau_N)\exp\left(\frac{-2\tau_H}{T_2'(H)}\right)\exp\left(\frac{-2\tau_N}{T_2'(N)}\right). \qquad (2)$$

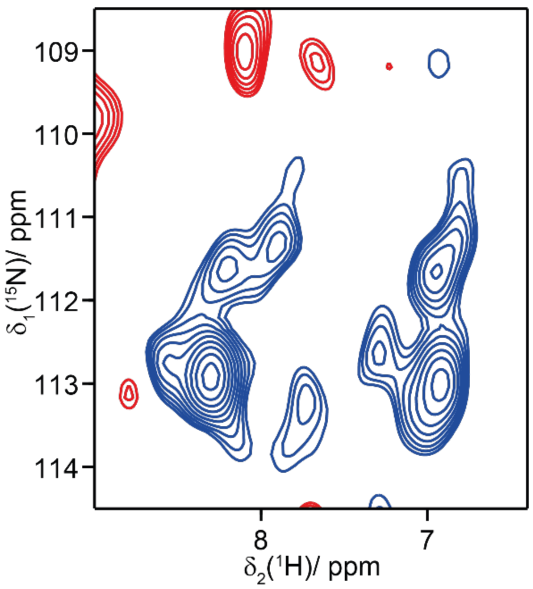

Figure S5: *Asparagine and glutamine side-chains in DnaB identified in INEPT experiments.* Difference spectrum of the spectra shown in Figure 3d. Positive contour levels representing asparagine and glutamine side-chain resonances are shown in blue, negative contour levels in red.

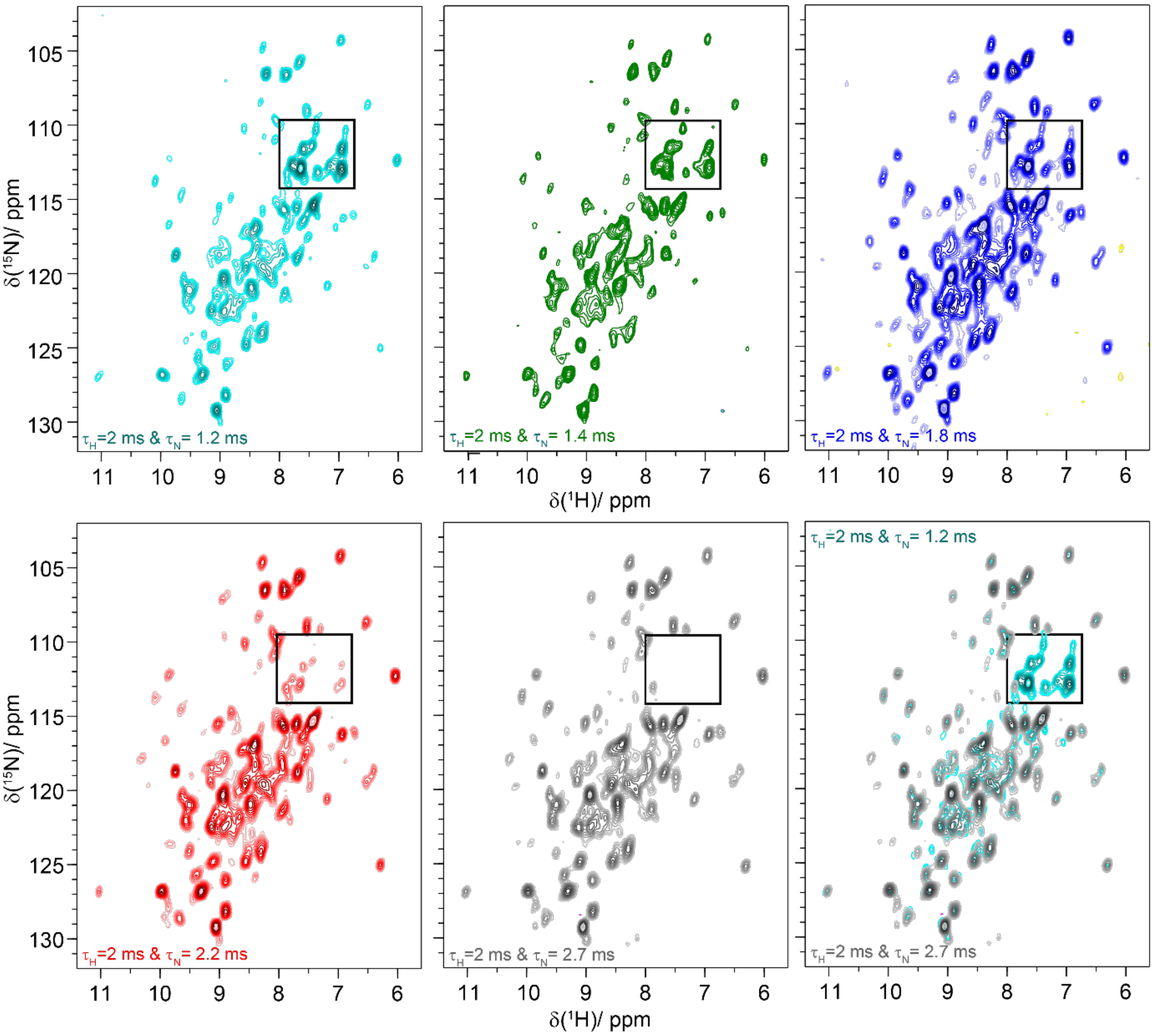

Figure S6: *Asparagine and glutamine side-chain filtering for DnaB.* $^{15}N,^{1}H$ refocused INEPT spectra for DnaB:ADP:AlF_4^-:DNA with varied τ_N mixing times. The intensity of the asparagine/glutamine side-chain resonances (highlighted by a black rectangle) are modified upon variation of the τ_N mixing times (see also Figure 3d).

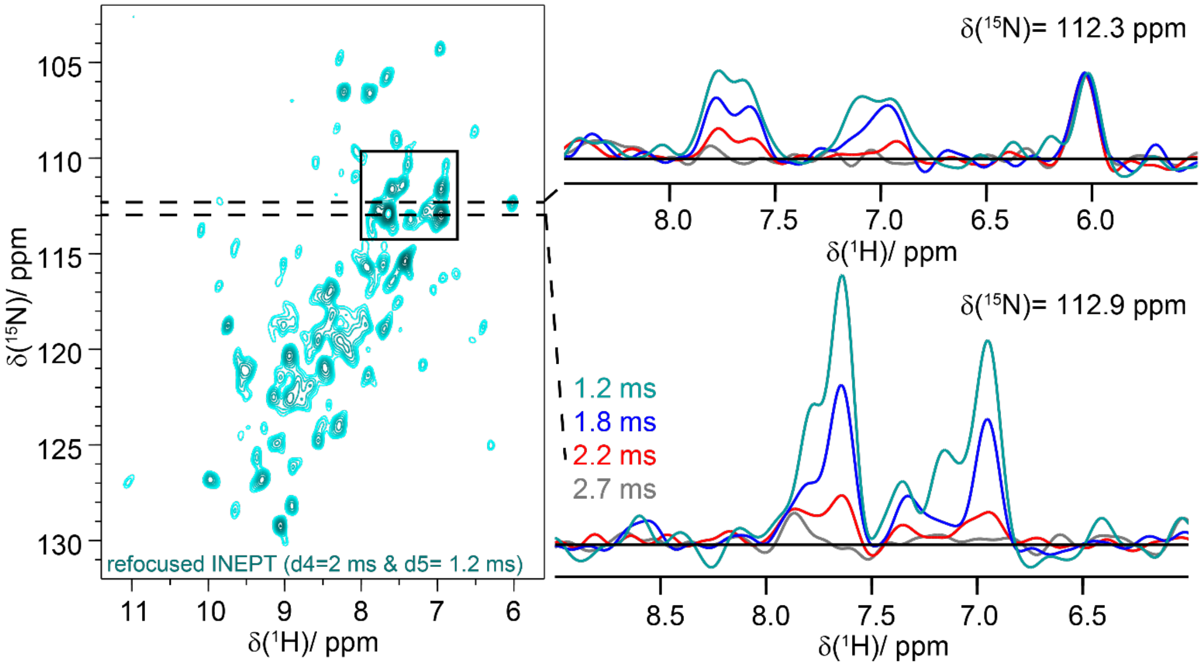

Figure S7: *Asparagine and glutamine side-chain filtering for DnaB.* Representative 1D traces along F2 of ^{15}N,^{1}H refocused INEPT spectra for DnaB:ADP:AlF_4^-:DNA with varied τ_N mixing times. The intensity of the asparagine/glutamine side-chain resonances (highlighted by a black rectangle) are modified upon variation of the τ_N mixing times (see also Figure 3d).

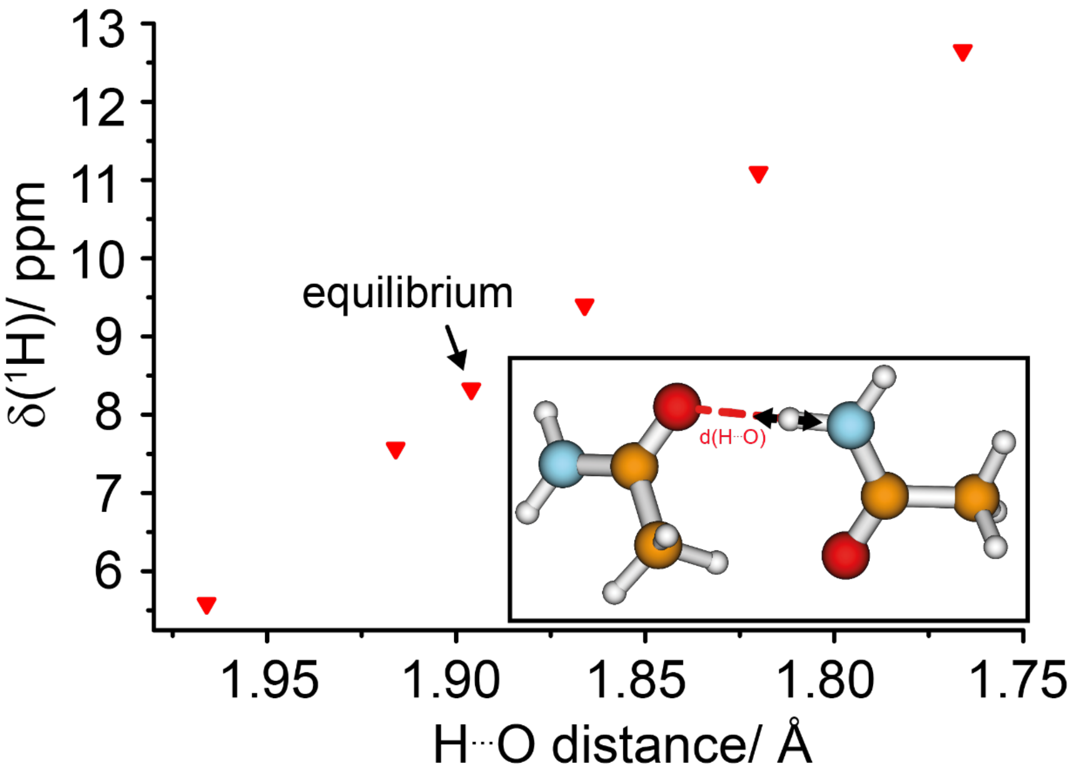

Figure S8: *The proton chemical shift is a sensitive tool to measure hydrogen bond strengths in asparagine ladders.* DFT calculations (B3-LYP/def2-TZVP) of ^{1}H chemical-shift values as a function of the hydrogen bond length in a model representing two asparagine side-chains. Only the position of the proton involved in the hydrogen bond is varied.

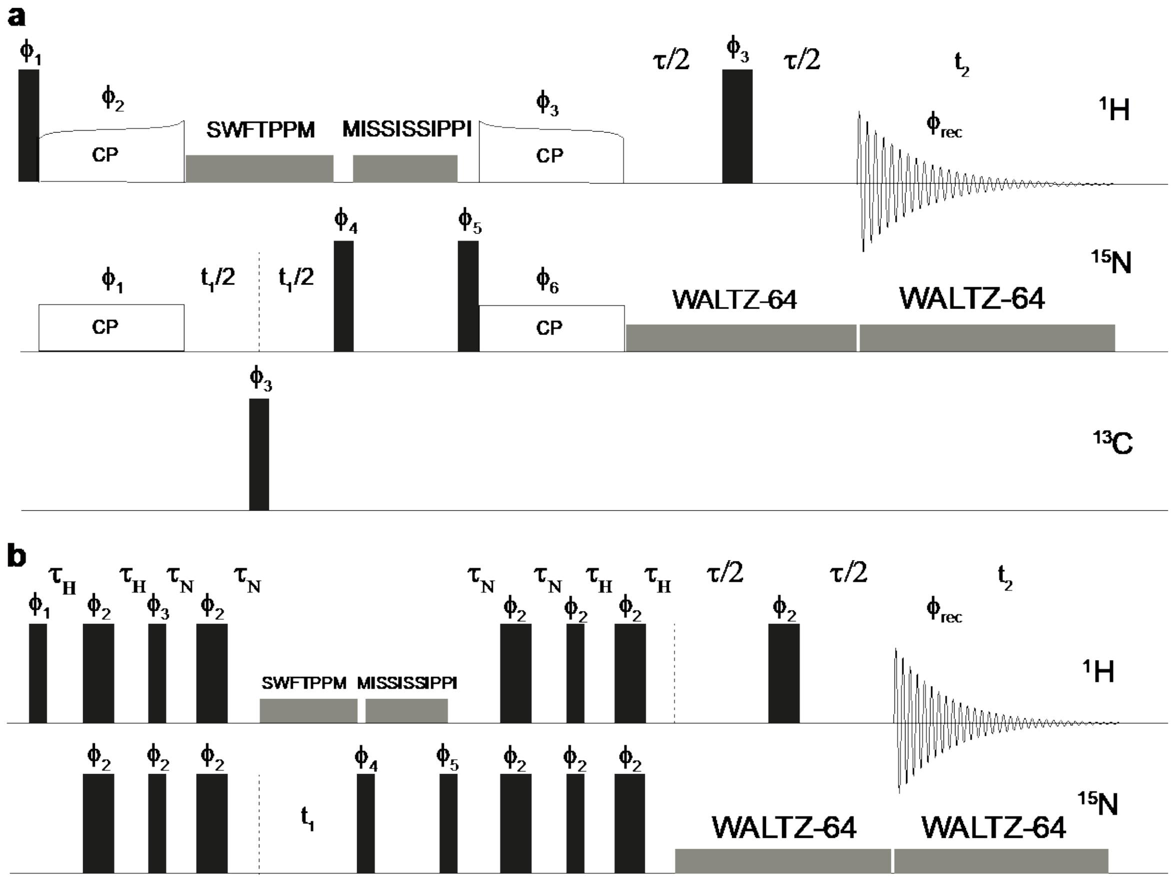

Figure S9: *Pulse schemes to measure T_2' relaxation times by solid-state NMR.* CP-based pulse sequence used to measure T_2' relaxation times (**a**) and INEPT-based sequence (**b**). The phase parameters in (**a**) correspond to: $\phi_1 = x\,x - x - x$, $\phi_2 = y$, $\phi_3 = x$, $\phi_4 = y$, $\phi_5 = y - y$, $\phi_6 = x$, $\phi_{\text{rec}} = x - x\,x - x$, while in (**b**) they correspond to: $\phi_1 = x\,x - x - x$, $\phi_2 = x$, $\phi_3 = y$, $\phi_4 = y$, $\phi_5 = y - y$, $\phi_{\text{rec}} = x - x\; - x\,x$.

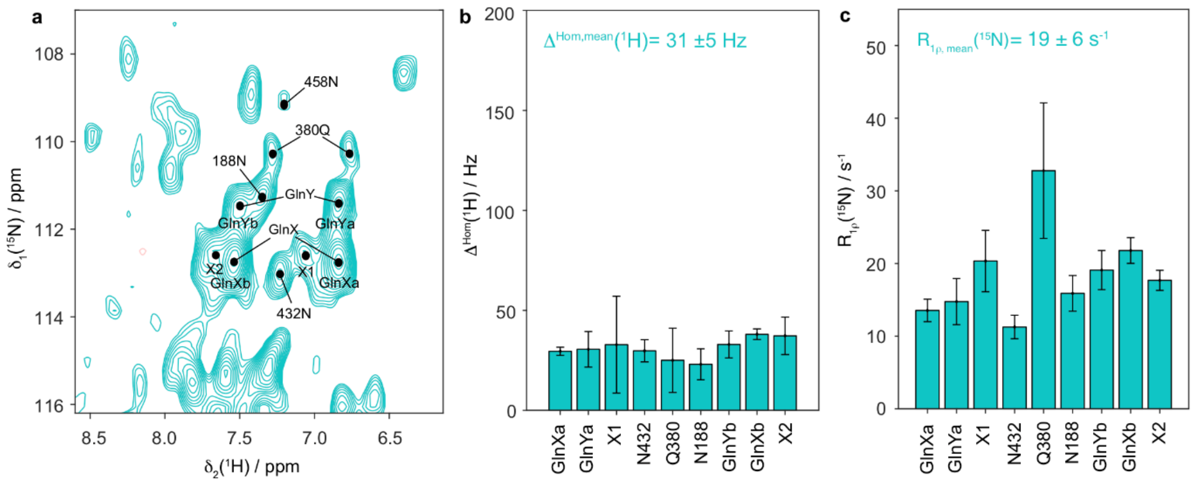

Figure S10: *Homogeneous proton linewidths and ^{15}N $R_{1\rho}$ rate constants of asparagine and glutamine side-chains in DnaB.* Zoom into the refocused INEPT-based spectrum of DUL DnaB (**a**) showing the asparagine and glutamine side-chain region and some resonance assignments. Homogeneous proton linewidths for asparagine and glutamine side-chains are given in **b**. Nitrogen spin-lock relaxation rate constants of asparagine and glutamine side-chains are shown in **c**.

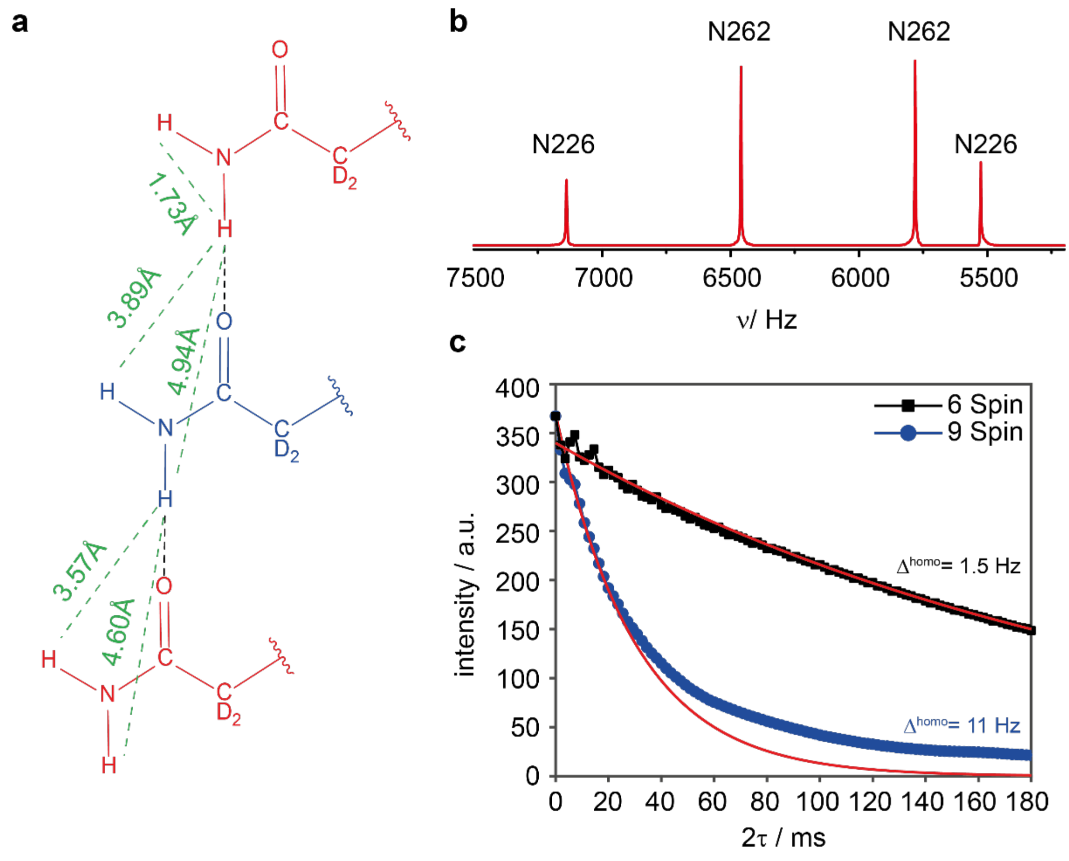

Figure S11: *Coherent effects to the proton linewidths of NH_2 protons in the asparagine ladder N226/N262 are negligible.* **a** Typical geometry of an asparagine ladder extracted from the pdb file 2KJ3 used in the numerical simulations. **b** Numerically simulated ^1H MAS spectrum of a six-spin system (the six proton spins shown in **a**). Only proton homonuclear dipolar couplings are considered. **c** Simulated spin echo decay profile for the geometry shown in **a** using a six-spin system (black) and a nine-spin system (six ^1H and three ^{15}N spins, blue). The red lines show exponential fits. For the nine spin system proton homonuclear dipolar couplings, ^1H-^{15}N heteronuclear dipolar and *J*-couplings are considered.

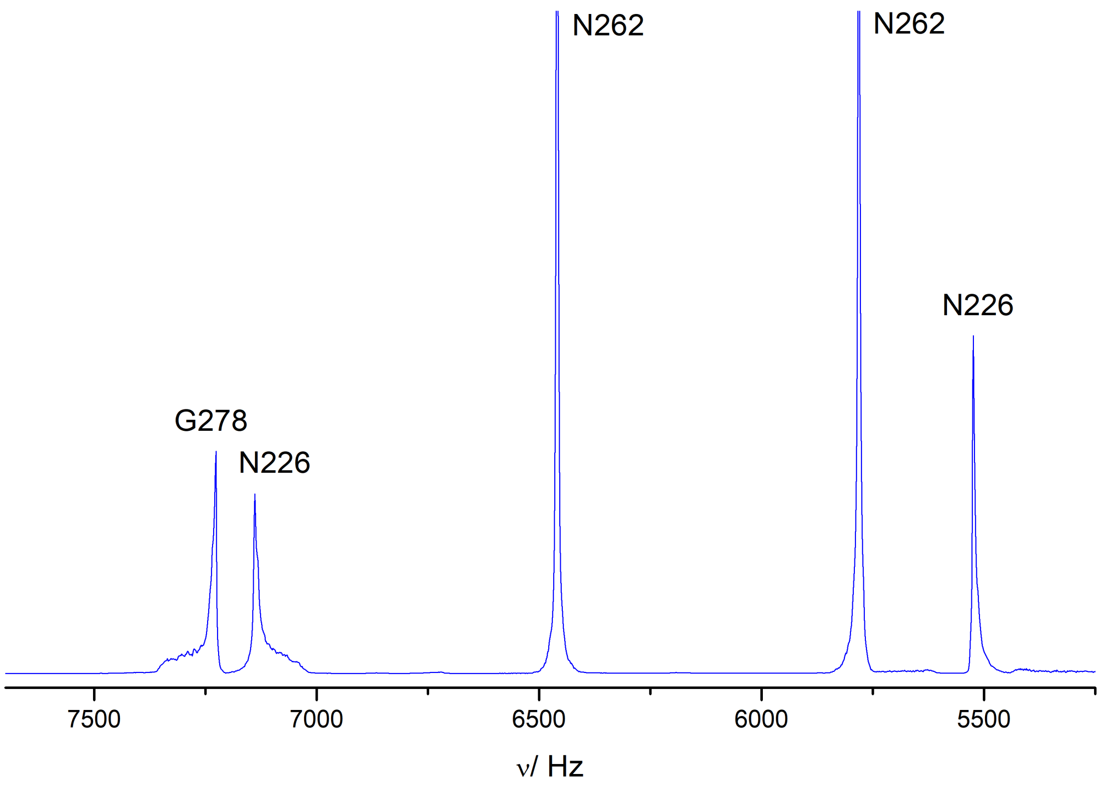

Figure S12: *Contribution of backbone amides to the proton linewidths of N side-chains.* Numerically simulated ^{1}H MAS spectrum of a seven-spin system (the six proton spins shown in Figure S11a and the amide backbone spin of G278 in the vicinity of N226 with distances of 2.64 and 3.43 Å to the protons of the N226 side-chains).

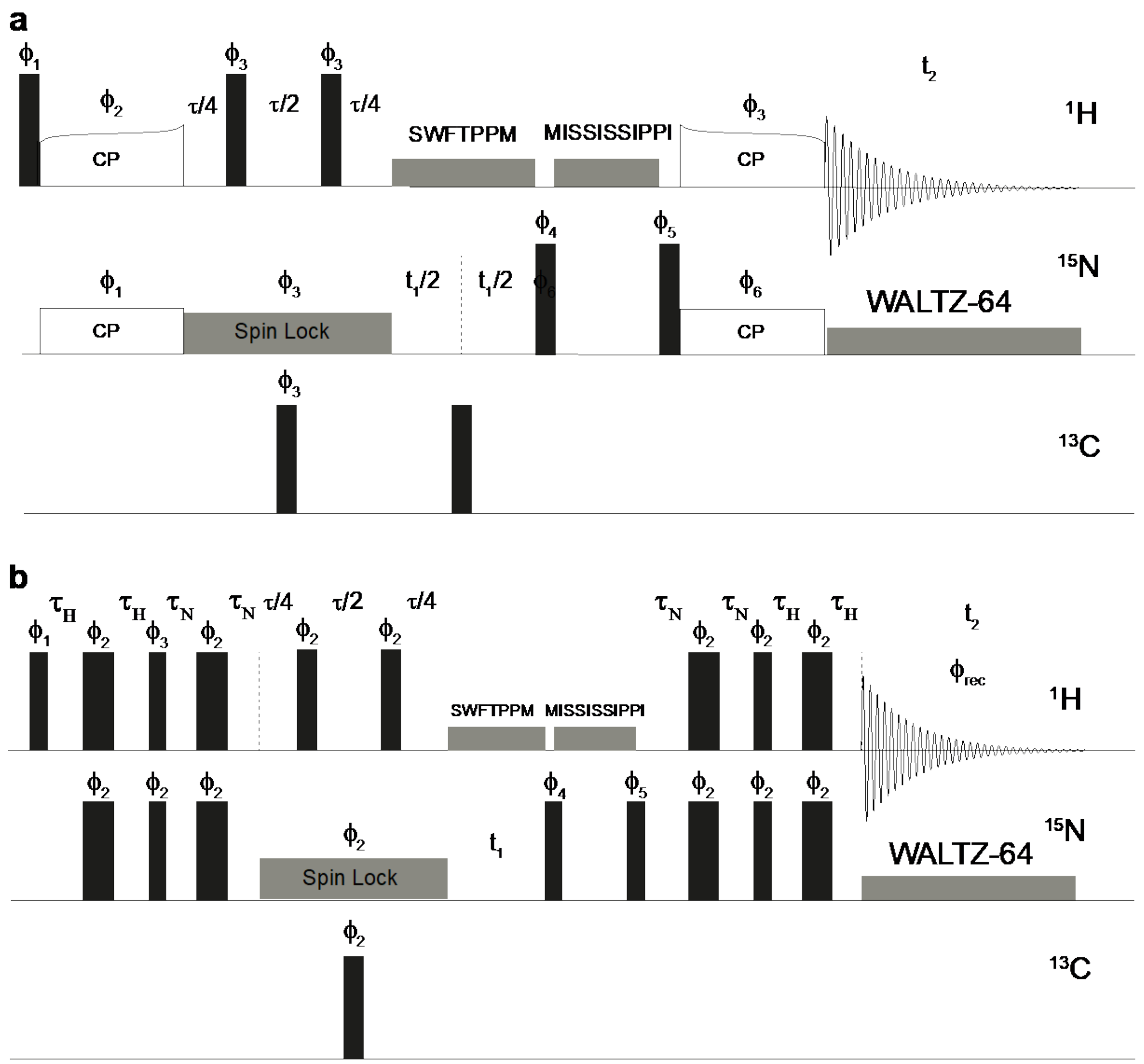

Figure S13: *Pulse schemes to measure ^{15}N $R_{1\rho}$ relaxation-rate constants by solid-state NMR.* CP-based pulse sequence (**a**) and INEPT-based sequence (**b**). The phase parameters in **(a)** correspond to: $\phi_1 = x\ x - x - x$, $\phi_2 = y$, $\phi_3 = x$, $\phi_4 = y$, $\phi_5 = y - y$, $\phi_6 = x$, $\phi_{\mathrm{rec}} = x - x\ x - x$, while in **(b)** they correspond to: $\phi_1 = x\ x - x - x$, $\phi_2 = x$, $\phi_3 = y$, $\phi_4 = y$, $\phi_5 = y - y$, $\phi_{\mathrm{rec}} = x - x\ - x\ x$.

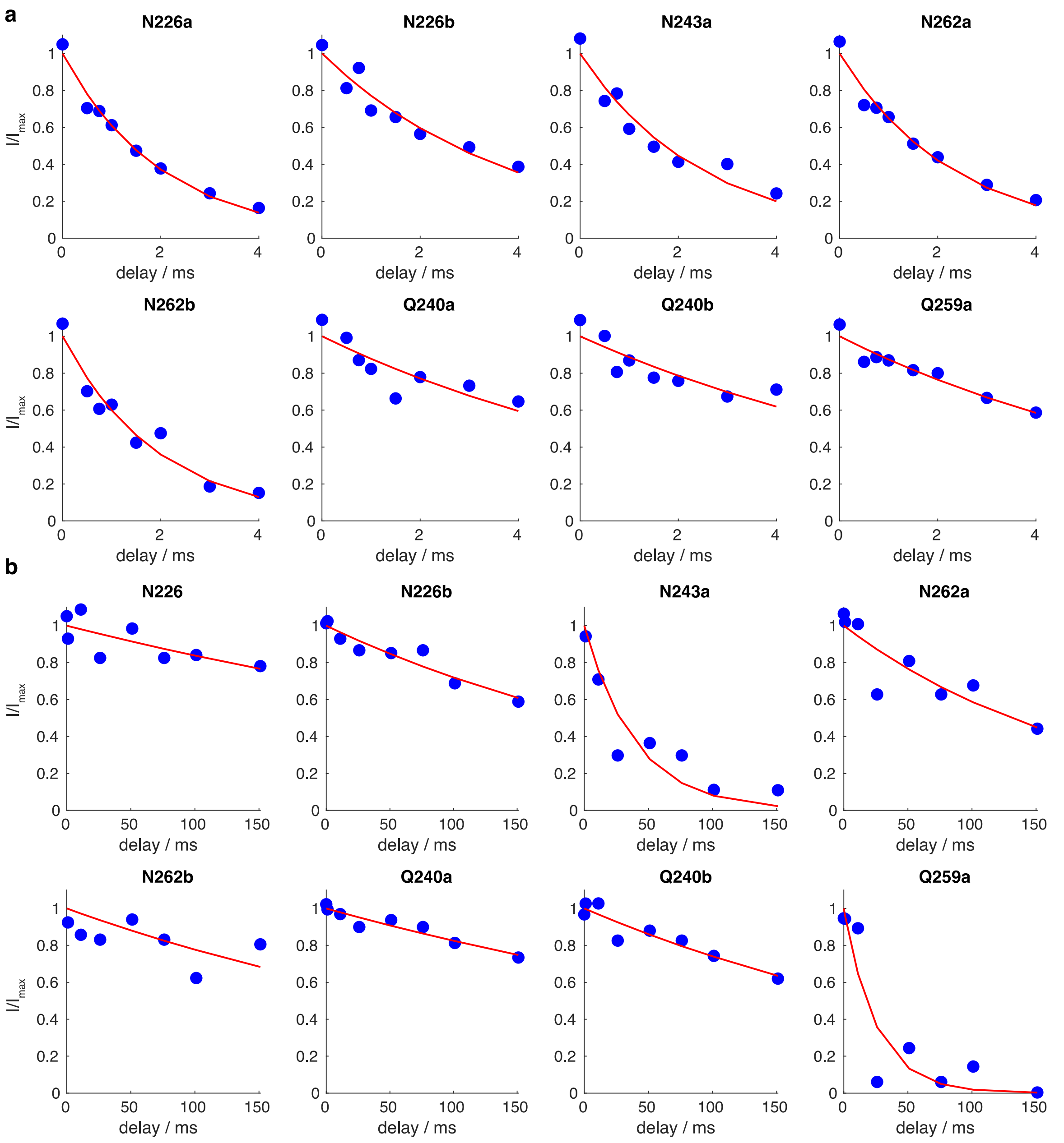

Figure S14: Site-specific decay curves (blue dots) and corresponding mono-exponential fits (red lines) for T_2'(^{1}H) **(a)** and $T_{1\rho}$(^{15}N) **(b)** relaxation experiments in DUL HET-s(218-289).

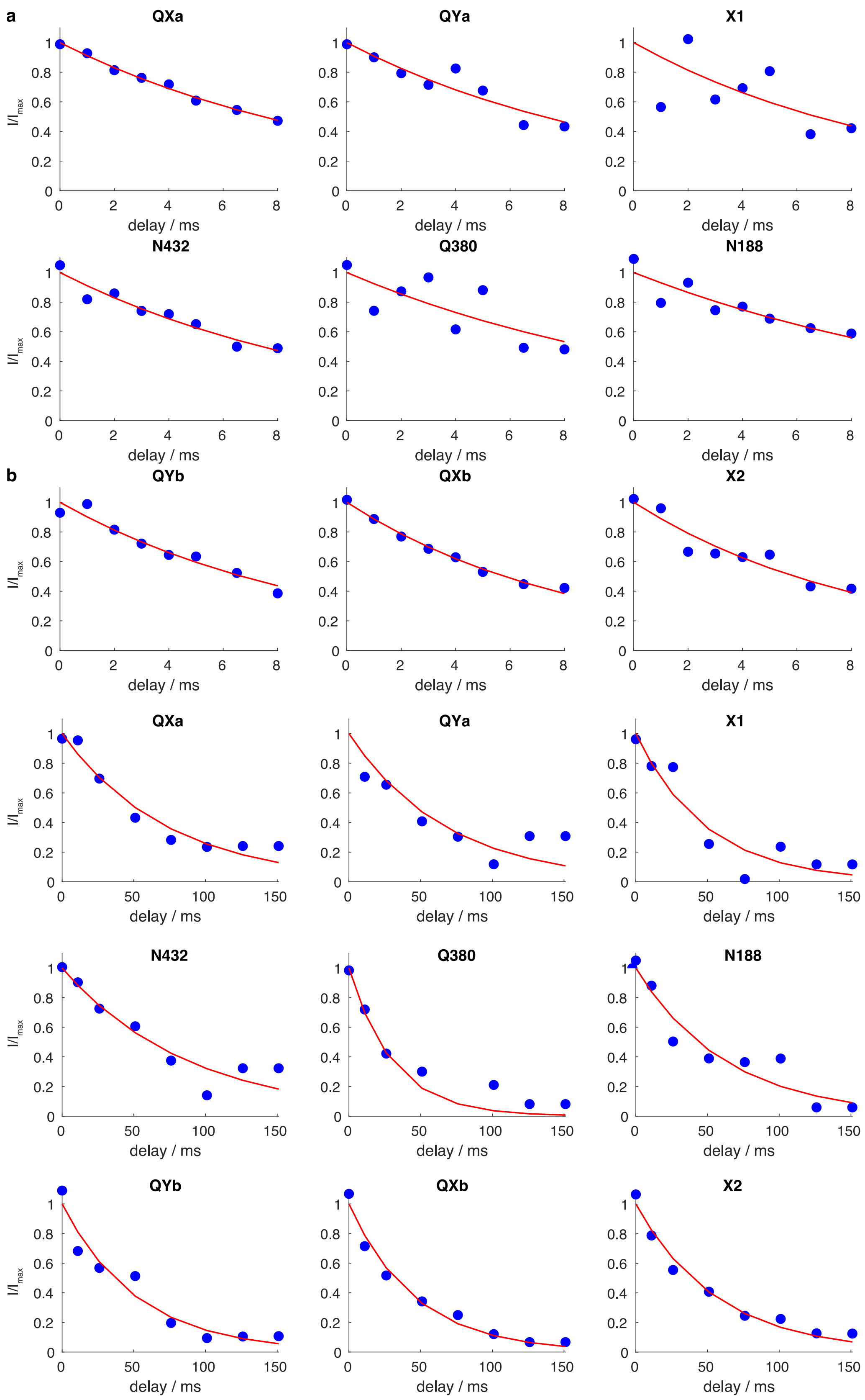

Figure S15: Site-specific decay curves (blue dots) and corresponding mono-exponential fits (red lines) for T_2'(^{1}H) **(a)** and $T_{1\rho}$(^{15}N) **(b)** relaxation experiments in DUL DnaB. For the numbering see Figure S10.

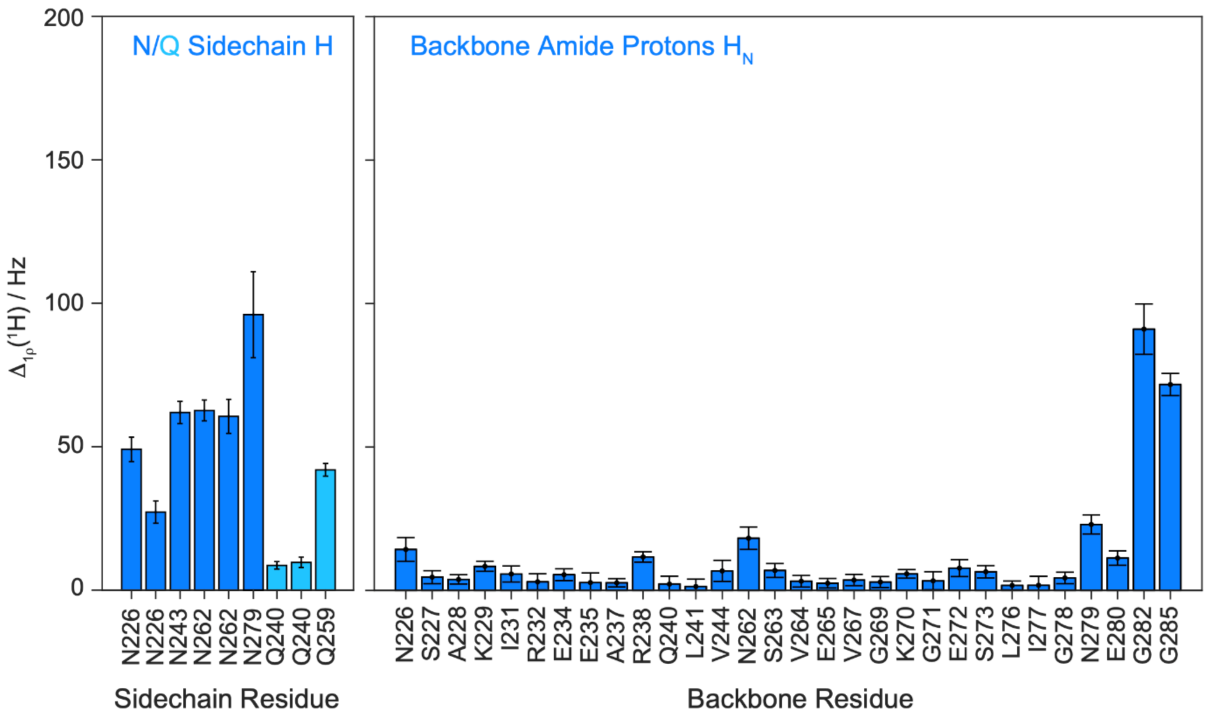

Figure S16: *Proton relaxation rate constants in the rotating frame expressed in frequency units of HET-s(218-289) fibrils.* The asparagine side-chain protons possess significantly larger values than rigid backbone amide protons.

Table S1: Overview about experimental solid-state NMR parameters used in the experiments.

**Experiment**	**CP-hNH (DUL HET-s (218-289))**
ν_r/ kHz	110
B_0/ T	20
**Transfer I**	HN-CP (ZQ)
ν_1(^{1}H) / kHz	137
ν_1(^{15}N) / kHz	33
INEPT delay τ_H / ms	Tangent ^{1}H
INEPT delay τ_N / ms	0.8
**Transfer II**	NH-CP (ZQ)
ν_1(^{1}H) / kHz	130
ν_1(^{15}N) / kHz	33
INEPT delay τ_H / ms	Tangent ^{1}H
INEPT delay τ_N / ms	1.2
^{1}H carrier/ ppm	4.8
^{15}N carrier/ ppm	117.5
t_1 increments	192
Sweep width (t_1)/ ppm	70
Acquisition time (t_1)/ ms	15.9
t_2 increments	2048
Sweep width (t_2)/ ppm	46.7
Acquisition time (t_2)/ ms	25.8
water Suppression	MISSISIPPI
ν_1(^{1}H) / kHz	20
Time water suppression / ms	120
^{15}N WALTZ64 decoupling power/ kHz	10
Interscan delay/ s	5
Number of scans	1.2
Measurement time/ h	32
ν_r/ kHz	2.0

**Experiment**	**INEPT-hNH (DUL HET-s (218-289))**	**INEPT-hNH (DUL HET-s (218-289))**
ν_r/ kHz	110	110
B_0/ T	20	20
**Transfer I**	HN-INEPT(refocused)	HN-INEPT(refocused)
$\nu_1(^1H)$ / kHz	150	150
$\nu_1(^{15}N)$ / kHz	62.5	62.5
INEPT delay τ_H / ms	2	2
INEPT delay τ_N / ms	1.2	2.7
**Transfer II**	NH- INEPT(refocused)	NH- INEPT(refocused)
$\nu_1(^1H)$ / kHz	150	150
$\nu_1(^{15}N)$ / kHz	62.5	62.5
INEPT delay τ_H / ms	2	2
INEPT delay τ_N / ms	1.2	2.7
^1H carrier/ ppm	4.8	4.8
^{15}N carrier/ ppm	117.5	117.5
t_1 increments	192	192
Sweep width (t_1)/ ppm	70	70
Acquisition time (t_1)/ ms	15.9	15.9
t_2 increments	8192	8192
Sweep width (t_2)/ ppm	46.7	46.7
Acquisition time (t_2)/ ms	103	103
water Suppression	MISSISIPPI	MISSISIPPI
$\nu_1(^1H)$ / kHz	20	20
Time water suppression / ms	120	120
^{15}N WALTZ64 decoupling power/ kHz	5	5
Interscan delay/ s	1	1
Number of scans	16	16
Measurement time/ h	0.9	0.9

**Experiment**	**hNCOH 3D DUL HET-s(218-289)**
ν_r/ kHz	110
B_0/ T	20
**Transfer I**	HN-CP (ZQ)
$\nu_1(^1H)$ / kHz	137
$\nu_1(^{15}N)$ / kHz	33
Shape	Tangent ^1H
Carrier / ppm	117.5
Time/ ms	1
**Transfer II**	NCO-CP (DQ)
$\nu_1(^{13}C)$ / kHz	75
$\nu_1(^{15}N)$ / kHz	33
Shape	Tangent ^{13}C
Carrier/ ppm	175
Time/ ms	20.0
**Transfer III**	COH-CP (DQ)
$\nu_1(^1H)$ / kHz	88
$\nu_1(^{13}C)$ / kHz	16
Shape	Tangent ^1H
Carrier/ ppm	4.8
Time/ ms	9
t_1 increments	50
Sweep width (t_1)/ ppm	45
Acquisition time (t_1)/ ms	6.4
t_2 increments	80
Sweep width (t_2)/ ppm	25
Acquisition time (t_2)/ ms	7.5
t_3 increments	2048
Sweep width (t_3)/ ppm	46.7
Acquisition time (t_3)/ ms	38.7
^1H swfTPPM decoupling/ kHz	10
^{15}N WALTZ64 decoupling/ kHz	5
^{13}C WALTZ64 decoupling/ kHz	5
MISSISSIPPI wat.supp./ kHz	20
Interscan delay/ s	1.0
Number of scans	48
Measurement time/ h	53

**Experiment**	**NCOCX 3D UL HET-s(218-289)**
ν_r/ kHz	17
B_0/ T	20
**Transfer I**	HN-CP (ZQ)
$\nu_1(^1H)$ / kHz	60
$\nu_1(^{15}N)$ / kHz	44
Shape	Tangent ^1H
Carrier / ppm	120
Time/ ms	1.2
**Transfer II**	NCO-CP (ZQ)
$\nu_1(^{13}C)$ / kHz	6
$\nu_1(^{15}N)$ / kHz	21
Shape	Tangent ^{13}C
Carrier/ ppm	176
Time/ ms	6.5
**Transfer III**	COCX-DARR
$\nu_1(^1H)$ / kHz	17
Shape	CW
Carrier/ ppm	176
Time/ ms	80
t_1 increments	60
Sweep width (t_1)/ ppm	50
Acquisition time (t_1)/ ms	7.0
t_2 increments	104
Sweep width (t_2)/ ppm	30
Acquisition time (t_2)/ ms	8.1
t_3 increments	2304
Sweep width (t_3)/ ppm	46
Acquisition time (t_3)/ ms	11.5
^1H SPINAL-64 decoupling/ kHz	90
Interscan delay/ s	2.8
Number of scans	16
Measurement time/ h	78

**Experiment**	**CP-hNH T_2'(1H) (DUL HET-s (218-289))**	**CP-hNH $T_{1\rho}$(15N) (DUL HET-s (218-289))**
v_r/ kHz	105	105
B_0/ T	20	20
**Transfer I**	HN-CP (DQ)	HN-CP (DQ)
$v_1(^1H)$ / kHz	85	84
$v_1(^{15}N)$ / kHz	15	15
Shape	Tangent ^1H	Tangent ^1H
Time / ms	2	2
**Transfer II**	NH-CP (DQ)	NH-CP (DQ)
$v_1(^1H)$ / kHz	85	84
$v_1(^{15}N)$ / kHz	15	15
Shape	Tangent ^1H	Tangent ^1H
Time / ms	2	2
**T_2'(^{1}H) Measurement**	Hahn-Echo block	-
**$T_{1\rho}$(15N) Measurement**	-	13 kHz Spin-Lock 15N
Relaxation delays / ms	0.001, 0.5, 0.75, 1, 1.5, 2, 3, 4	0.001, 1, 11, 26, 51, 76, 101, 151
^1H carrier/ ppm	4.7	4.7
^{15}N carrier/ ppm	117.5	117.5
t_1 increments	192	192
Sweep width (t_1)/ ppm	70	70
Acquisition time (t_1)/ ms	15.9	15.9
t_2 increments	2048	2048
Sweep width (t_2)/ ppm	40	40
Acquisition time (t_2)/ ms	30.0	30.0
Water Suppression	MISSISIPPI	MISSISIPPI
$v_1(^1H)$ / kHz	20	10
Time / ms	120	120
^1H swfttpm decoupling power/ kHz	10	10
^{15}N WALTZ64 decoupling power/ kHz	5	10
Interscan delay/ s	1	2
Number of scans	108	88
Measurement time/ h	46	75

Experiment	INEPT-hNH T_2'(^{1}H) (DUL DnaB ADP:AlF$_4^-$:ssDNA)	INEPT-hNH $T_{1\rho}$(^{15}N) (DUL DnaB ADP:AlF$_4^-$:ssDNA)
ν_r/ kHz	110	110
B_0/ T	20	20
**Transfer I**	HN-INEPT(refocused)	HN-INEPT(refocused)
ν_1(^{1}H) / kHz	150	150
ν_1(^{15}N) / kHz	62.5	62.5
INEPT delay τ_H / ms	2	2
INEPT delay τ_N / ms	1.2	1.2
**Transfer II**	NH- INEPT(refocused)	NH- INEPT(refocused)
ν_1(^{1}H) / kHz	150	150
ν_1(^{15}N) / kHz	62.5	62.5
INEPT delay τ_H / ms	2	2
INEPT delay τ_N / ms	1.2	1.2
**T_2'(^{1}H) Measurement**	Hahn-Echo block	-
**$T_{1\rho}$(15N) Measurement**	-	13 kHz Spin-Lock ^{15}N
Relaxation delays / ms	0.002, 1, 2, 3, 4, 5, 6.5, 8	0.001, 11, 26, 51, 76, 101, 126, 151
^{1}H carrier/ ppm	4.8	4.8
^{15}N carrier/ ppm	117.5	117.5
t_1 increments	192	192
Sweep width (t_1)/ ppm	70	70
Acquisition time (t_1)/ ms	15.9	15.9
t_2 increments	8192	8192
Sweep width (t_2)/ ppm	46.7	46.7
Acquisition time (t_2)/ ms	103	103
Water Suppression	MISSISIPPI	MISSISIPPI
ν_1(^{1}H) / kHz	20	20 kHz
Time / ms	120	120 m
^{15}N WALTZ64 decoupling power/ kHz	5	5
Interscan delay/ s	1	2
Number of scans	104	40
Measurement time/ h	44	34

**Experiment**	**CP-hNH $T_{1\rho}$(1H) (DUL HET-s (218-289))**
ν_r/ kHz	105
B_0/ T	20
**Transfer I**	HN-CP (DQ)
$\nu_1(^1H)$ / kHz	80
$\nu_1(^{15}N)$ / kHz	16
Shape	Tangent ^1H
Time / ms	1.2
**Transfer II**	NH-CP (DQ)
$\nu_1(^1H)$ / kHz	80
$\nu_1(^{15}N)$ / kHz	16
Shape	Tangent ^1H
Time / ms	1.2
**$T_{1\rho}$(15N) Measurement**	13 kHz Spin-Lock 1H
Relaxation delays / ms	0.001, 1, 2, 3, 4, 6, 8, 10
^1H carrier/ ppm	4.8
^{15}N carrier/ ppm	117.5
t_1 increments	192
Sweep width (t_1)/ ppm	70
Acquisition time (t_1)/ ms	15.9
t_2 increments	2048
Sweep width (t_2)/ ppm	46.7
Acquisition time (t_2)/ ms	25.8
Water Suppression	MISSISIPPI
$\nu_1(^1H)$ / kHz	20
Time / ms	120
^1H swfttpm decoupling power/ kHz	10
^{15}N WALTZ64 decoupling power/ kHz	5
Interscan delay/ s	1.2
Number of scans	96
Measurement time/ h	56

SIMPSON simulation code

a) 6-spin system

```
spinsys {
channels 1H
nuclei 1H 1H 1H 1H 1H 1H
shift 1 7.6p 0p 0  0 0 0
shift 2 6.8p 0p 0  0 0 0
shift 3 8.4p 0p 0  0 0 0
shift 4 6.5p 0p 0  0 0 0
shift 5 7.6p 0p 0  0 0 0
shift 6 6.8p 0p 0  0 0 0
dipole 1 2 -23113.640421904358 0 111.29274517320874 157.86895025570306
dipole 1 3 -997.953558489671 0 21.528630248401992 -64.99075319104256
dipole 1 4 -2037.784990682943 0 20.931861103235338 -116.62033379879665
dipole 1 5  -138.56251683618703 0 23.231319392713846 -63.50308769682698
dipole 1 6  -201.03186504572486 0 18.032691450463528 -82.83956610617709
dipole 2 3  -524.2650023279742 0 31.417958215722564 -44.860283909284696
dipole 2 4 -1135.4647420291858 0 25.63441156966358 -64.77503096552351
dipole 2 5 -98.62139832887591 0 28.436050194738637 -51.394743336778795
dipole 2 6 -145.17654974318248 0 23.058432914120672 -60.332432791214224
dipole 3 4 -23045.02637464897 0 123.54030206060665 163.97294337719302
dipole 3 5 -1231.9577757412205 0 25.071388433031224 -62.12107218449644
dipole 3 6 -2636.4650791486047 0 16.97637936399455 -115.01686826357206
dipole 4 5 -554.0907894407011 0 31.410069620607445 -42.69594216973688
dipole 4 6 -1177.1601802062062 0 20.60714005704576 -54.802503177592136
dipole 5 6 -23066.066257032304 0 115.74854815891698 150.06849946060154
}

par {
        spin_rate               110000
        crystal_file            rep2000
        np                              65536
        gamma_angles                    1
        proton_frequency        850e6
        start_operator          Inx
        detect_operator         Inp
        sw                              spin_rate
        variable zf                     np*2
        variable dw                     1e6/sw
        method                                  gcompute
        num_cores                       1
        verbose                                 1101

}


proc pulseq {} {
  global par

  acq_block {
```

```
   delay $par(dw)
         }
}


proc main {} {
         global par
         set f [fsimpson]
         fzerofill $f 32768
         fsave $f $par(name).fid -xreim
         faddlb $f 0 0
         fft $f
         fsave $f $par(name).spe
}
```

b) 7-spin system

```
spinsys {
channels 1H
nuclei 1H 1H 1H 1H 1H 1H 1H
shift 1 7.6p 0p 0  0 0 0
shift 2 6.8p 0p 0  0 0 0
shift 3 8.4p 0p 0  0 0 0
shift 4 6.5p 0p 0  0 0 0
shift 5 7.6p 0p 0  0 0 0
shift 6 6.8p 0p 0  0 0 0
shift 7 8.5p 0p 0  0 0 0
dipole 1 2 -23113.640421904358 0 111.29274517320874 157.86895025570306
dipole 1 3 -997.953558489671 0 21.528630248401992 -64.99075319104256
dipole 1 4 -2037.784990682943 0 20.931861103235338 -116.62033379879665
dipole 1 5  -138.56251683618703 0 23.231319392713846 -63.50308769682698
dipole 1 6  -201.03186504572486 0 18.032691450463528 -82.83956610617709
dipole 2 3  -524.2650023279742 0 31.417958215722564 -44.860283909284696
dipole 2 4 -1135.4647420291858 0 25.63441156966358 -64.77503096552351
dipole 2 5 -98.62139832887591 0 28.436050194738637 -51.394743336778795
dipole 2 6 -145.17654974318248 0 23.058432914120672 -60.332432791214224
dipole 3 4 -23045.02637464897 0 123.54030206060665 163.97294337719302
dipole 3 5 -1231.9577757412205 0 25.071388433031224 -62.12107218449644
dipole 3 6 -2636.4650791486047 0 16.97637936399455 -115.01686826357206
dipole 4 5 -554.0907894407011 0 31.410069620607445 -42.69594216973688
dipole 4 6 -1177.1601802062062 0 20.60714005704576 -54.802503177592136
dipole 5 6 -23066.066257032304 0 115.74854815891698 150.06849946060154
dipole 7 1 -676.2515637004187 0 134.58534966182435 58.21155252033565
dipole 7 2 -525.278079658127 0 138.42910533050105 81.29680117218014
dipole 7 3 -2968.987981237454 0 79.13674162277177 31.488388840263465
dipole 7 4 -6527.172537682783 0 96.76490071080362 55.473473699320984
dipole 7 5 -522.3244080023001 0 38.18062032728285 0.5597872862781581
dipole 7 6 -1082.0713998953788 0 32.28608804491319 18.533761135418647
}
```

```
par {
	spin_rate		110000
	crystal_file		rep2000
	np			65536
	gamma_angles		1
	proton_frequency	850e6
	start_operator		Inx
	detect_operator		Inp
	sw			spin_rate
	variable zf		np*2
	variable dw		1e6/sw
	method			gcompute
	num_cores		1
	verbose			1101

}


proc pulseq {} {
  global par

  acq_block {
   delay $par(dw)
	}
}


proc main {} {
	global par
	set f [fsimpson]
	fzerofill $f 32768
	fsave $f $par(name).fid -xreim
	faddlb $f 0 0
	fft $f
	fsave $f $par(name).spe

}
```

Gamma simulation code

```
----------------------- input file 1 each rotor period ------------------------------
./mas_echo 6spin_HH.sys\
          0      -46227          0      111.29       157.87\
          0     -1995.9          0      21.529      -64.991\
          0     -4075.6          0      20.932      -116.62\
          0     -277.13          0      23.231      -63.503\
          0     -402.06          0      18.033       -82.84\
          0     -1048.5          0      31.418       -44.86\
          0     -2270.9          0      25.634      -64.775\
          0     -197.24          0      28.436      -51.395\
          0     -290.35          0      23.058      -60.332\
          0      -46090          0      123.54       163.97\
          0     -2463.9          0      25.071      -62.121\
          0     -5272.9          0      16.976      -115.02\
          0     -1108.2          0       31.41      -42.696\
          0     -2354.3          0      20.607      -54.803\
          0      -46132          0      115.75       150.07\
              0.30 0.0 0.00 0 0 0\
             -0.50 0.0 0.00 0 0 0\
              1.10 0.0 0.00 0 0 0\
             -0.80 0.0 0.00 0 0 0\
              0.30 0.0 0.00 0 0 0\
             -0.50 0.0 0.00 0 0 0\
              850 7 100 110000 1 100 mas6AA03a >& mas6AA03a.log
```

```
------------------------- mas_echo.cc ---------------------------------------------
/*

mas_echo.cc

Simulation of homonuclear dipolar coupled spin system.
Can run up to 10 spins with the CSA tensors.
Brute force integration of the MAS rotation (one cycle)
added isotropic chemical shifts

Echo with ideal 180 degree pulse.

*/

#include "gamma.h"
#include <sys/time.h>
#include <sys/resource.h>

#define NPROP 1000
#define MAXSPINS 10

using namespace std;
```

```
int main(int argc, char *argv[])

{
  spin_system ax;
  gen_op Hrf,Ham, U, Up, H[5], sigma, sigma1, detect;
  spin_T  Hdip[MAXSPINS][MAXSPINS];
  space_T Adip[MAXSPINS][MAXSPINS], Adip_R[MAXSPINS][MAXSPINS];
  space_T Acsa[MAXSPINS], Acsa_R[MAXSPINS];
  double D[MAXSPINS][MAXSPINS];
  double J[MAXSPINS][MAXSPINS];
  double iso_CSA[MAXSPINS];
  double eta_CSA[MAXSPINS];
  double delta_CSA[MAXSPINS];
  int i,j,k,Fnp,count,qu,steps,nrotor,necho;
  string name, names;
  const double thetam=54.73561032;
  double nu1h, mas_freq;
  double ltime, time, time_prop, time_mas, scale;
  int nspins;
  double alpha,beta,gamma;
  double alpha_CSA[MAXSPINS],beta_CSA[MAXSPINS];
  double gamma_CSA[MAXSPINS];
  double alpha_D[MAXSPINS][MAXSPINS],beta_D[MAXSPINS][MAXSPINS];
  double gamma_D[MAXSPINS][MAXSPINS];
  struct rusage me;

  int value1[] = {1, 50, 100, 144, 200, 300, 538, 1154, 2000, 5000, 10000, 50000, 100000};
  int value2[] = {1,  7,  27,  11,  29,  37,  55,  107,  297, 1197,  3189, 14857,  38057};
  int value3[] = {1, 11,  41,  53,  79,  61, 229,  271,  479, 1715,  4713,  9027,  27205};

  count = 1;
  query_parameter(argc,argv,count++,"Spin System Name            ? ", names);
//setup for the spin system
  ax.read(names);
  nspins = ax.spins();
  if(nspins < 2 || nspins >= 10)
  { cerr << "This program is written for a two to nine spin system.\n";
    cerr << "Please change your spin system definition in \n";
    cerr << "the file " << names << ".\n";
    cerr << "Aborting ....\n\n";
    exit(-1);
  }
// !!!!!!!!!!!!!!!!!!!!!!!!!!!!!!!!!!!!!!!!!!!!!!!!
// this is -delta/2Pi = omegaD/Pi = +mu/4Pi gamma1*gamma2*hbar/Pi /r^3
// !!!!!!!!!!!!!!!!!!!!!!!!!!!!!!!!!!!!!!!!!!!!!!!!
  for(i=0;i<nspins-1;++i)
  { for(j=i+1;j<nspins;++j)
    { query_parameter(argc,argv,count++,"Dipolar Coupling Constant   ? ", J[i][j]);
      query_parameter(argc,argv,count++,"Dipolar Coupling Constant   ? ", D[i][j]);
      query_parameter(argc,argv,count++,"Euler angle alpha           ? ", alpha_D[i][j]);
      query_parameter(argc,argv,count++,"Euler angle beta            ? ", beta_D[i][j]);
```

```
    query_parameter(argc,argv,count++,"Euler angle gamma          ? ", gamma_D[i][j]);
   }
  }
  for(i=0;i<nspins;++i)
  { query_parameter(argc,argv,count++,"Isotropic chemical shift    ? ", iso_CSA[i]);
   query_parameter(argc,argv,count++,"anisotropy chemical shift   ? ", delta_CSA[i]);
   query_parameter(argc,argv,count++,"asymmetry chemical shift    ? ", eta_CSA[i]);
   query_parameter(argc,argv,count++,"Euler angle alpha          ? ", alpha_CSA[i]);
   query_parameter(argc,argv,count++,"Euler angle beta           ? ", beta_CSA[i]);
   query_parameter(argc,argv,count++,"Euler angle gamma          ? ", gamma_CSA[i]);
  }
  query_parameter(argc,argv,count++,"Proton frequency           ? ", nu1h);
  query_parameter(argc,argv,count++,"Powder Quality (cheng)      ? ", qu);
  query_parameter(argc,argv,count++,"Number of time steps        ? ", steps);
  query_parameter(argc,argv,count++,"spinning speed             ? ", mas_freq);
  query_parameter(argc,argv,count++,"Number of rotor periods     ? ", nrotor);
  query_parameter(argc,argv,count++,"Number of echo periods      ? ", necho);
  query_parameter(argc,argv,count++,"Output Filename            ? ", name);

  Fnp = necho;

  time_mas  = (1.0/mas_freq);
  time_prop  = nrotor*(1.0/mas_freq);
  time       = (1.0/mas_freq)/steps;

  for(k=0;k<nspins;++k)
  { iso_CSA[k]=nu1h*iso_CSA[k];
   delta_CSA[k]=nu1h*delta_CSA[k];
  }

  cout << "\n\nSimulation of isotropic chemical shift by dipolar coupling\n";
  cout <<
"============================================================\n\n";
  cout << "Program version: " << __FILE__ << " compiled at " << __DATE__ ", "
      << __TIME__ << "\n\n";
  cout << "Parameters:\n";
  cout << "rotation angle thetam         : " << thetam << " Degree\n";
  cout << "size of spin system           : " << nspins << " spins\n";
  for(i=0;i<nspins-1;++i)
  { for(j=i+1;j<nspins;++j)
   { cout << "J       coupling constant (" << i << "," << j << ") : " <<
           J[i][j] << " Hz\n";
    cout << "dipolar coupling constant (" << i << "," << j << ") : " <<
           D[i][j] << " Hz\n";
    cout << "relativ orientation of D tensor: (" << alpha_D[i][j] << "," <<
            beta_D[i][j] << "," << gamma_D[i][j] << ")\n";
   }
  }
  for(i=0;i<nspins;++i)
  { cout << "isotropic chemical shift (" << i << ") : " << iso_CSA[i] << " Hz\n";
   cout << "anisotropy chemical shift (" << i << "): " << delta_CSA[i] << " Hz\n";
```

```
    cout << "asymmetry chemical shift (" << i << ") : " << eta_CSA[i] << "\n";
    cout << "relativ orientation of CSA tensor: (" << alpha_CSA[i] << "," <<
            beta_CSA[i] << "," << gamma_CSA[i] << ")\n";
  }
  cout << "# of rotor periods:            " << nrotor << "\n";
  cout << "Powder Quality Number:         " << qu << "  (" << value1[qu] <<
         " orientations)\n";
  cout << "Number of data points:         " << Fnp << " points\n";
  cout << "MAS frequency:                 " << mas_freq << " Hz\n";
  cout << "time increments:               " << time << "s\n";
  cout << "Output filename:               " << name << "\n";
  cout << "\n";
  cout.flush();

  block_1D data(Fnp);

//setup for the hamiltonian
  for(i=0;i<nspins-1;++i)
  { for(j=i+1;j<nspins;++j)
    { Hdip[i][j] = T_D(ax,i,j);
    }
  }

//setup for the space tensor
  matrix help(3,3,0);
  for(i=0;i<nspins-1;++i)
  { for(j=i+1;j<nspins;++j)
    { help.put_h(-1.0/2.0,0,0);
      help.put_h(-1.0/2.0,1,1);
      help.put_h( 1.0,2,2);
      help   = - (complex) D[i][j] * help;
      Adip[i][j] = A2(help);
      Adip[i][j] = Adip[i][j].rotate(alpha_D[i][j],beta_D[i][j],gamma_D[i][j]);
    }
  }

  for(i=0;i<nspins;++i)
  { help.put(-1.0/2.0*(1.0+eta_CSA[i]),0,0);
    help.put(-1.0/2.0*(1.0-eta_CSA[i]),1,1);
    help.put( 1.0,2,2);
    help = (complex) delta_CSA[i] * help;
    Acsa[i] = A2(help);
    Acsa[i] = Acsa[i].rotate(alpha_CSA[i],beta_CSA[i],gamma_CSA[i]);
  }

  string name1 = name+".mat";
  string name2 = name;

//here starts the powder loop
//reference JCP 59 (8), 3992 (1973).
```

```
  for(count=1; count<=value1[qu]; ++count)
  { beta  = 180.0 * count/value1[qu];
    alpha = 360.0 * ((value2[qu]*count) % value1[qu])/value1[qu];
    gamma = 360.0 * ((value3[qu]*count) % value1[qu])/value1[qu];
    if(count % 100 == 1)
    { getrusage(0, & me);
      cout << count << "\tbeta = " << beta << "\talpha = "
           << alpha << "\tgamma = " << gamma
           << ",\ttime used: " << me.ru_utime.tv_sec << " seconds\n";
      cout.flush();
    }
    scale = sin(beta/180.0*PI);

    detect=  Fm(ax,"1H");

//now we rotate the space tensor
    for(i=0;i<nspins;++i)
    { for(j=i+1;j<nspins;++j)
      { Adip_R[i][j] = Adip[i][j].rotate(alpha,beta,gamma);
      }
    }
    for(i=0;i<nspins;++i)
    { Acsa_R[i] = Acsa[i].rotate(alpha,beta,gamma);
    }

//zero all components
    for(i=0;i<5;++i)
      H[i] = gen_op();

//this is the dipolar part
    for(i=0;i<nspins;++i)
    { H[2] += iso_CSA[i]*Iz(ax,i);
    }
    for(k=-2;k<=2;++k)
    { for(i=0;i<nspins;++i)
      { H[k+2] += Acsa_R[i].component(2,k) * d2(k,0,thetam)*2.0/sqrt(6.0)*Iz(ax,i);
        for(j=i+1;j<nspins;++j)
        { if(ax.isotope(i) != ax.isotope(j))
          { H[k+2] += Adip_R[i][j].component(2,k) * d2(k,0,thetam) *
1.0/sqrt(6.0)*2*Iz(ax,i)*Iz(ax,j);
          }
          else
          { H[k+2] += Adip_R[i][j].component(2,k) * d2(k,0,thetam) *
Hdip[i][j].component(2,0);
          }
        }
      }
    }

    U = Ie(ax,0);
```

```
//now we calculate the propagator over one cycle of the MAS
    for(ltime=0.5*time;ltime<=steps*time;ltime += time)
    { Ham = gen_op();
      for(i=-2;i<=2;++i)
        Ham += exp(complex(0,i*2.0*PI*ltime*mas_freq)) * H[i+2];
      U &= prop(Ham,time);
    }
    Up = pow(U,nrotor);
    sigma =  Fx(ax,"1H");
    sigma.set_DBR();
    sigma1 = sigma;
    for(i=0;i<Fnp;++i)
    { data(i) += proj(sigma1,detect)*scale;
      sigma.sim_trans_ip(Up);
      sigma1 = Ixypuls(ax,sigma,"1H",0,180);
      for(j=0;j<=i;++j)
      { sigma1.sim_trans_ip(Up);
      }
    }
  } // end of powder loop
  MATLAB(name1,name2,data,1);
  exit(0);
}
```

References

1. Takegoshi, K., Nakamura, S. & Terao, T. 13C–1H dipolar-assisted rotational resonance in magic-angle spinning NMR. *Chem. Phys. Lett.* **344**, 631-637 (2001).
2. Takegoshi, K., Nakamura, S. & Terao, T. 13C–13C polarization transfer by resonant interference recoupling under magic-angle spinning in solid-state NMR. *Chem. Phys. Lett.* **307**, 295-302 (1999).
3. Wasmer, C., Lange, A., Van Melckebeke, H., Siemer, A.B., Riek, R. & Meier, B.H. Amyloid Fibrils of the HET-s(218-289) Prion Form a β Solenoid with a Triangular Hydrophobic Core. *Science (Washington, DC, U. S.)* **319**, 1523-1526 (2008).
4. Pegg, D.T., Doddrell, D.M., Brooks, W.M. & Robin Bendall, M. Proton polarization transfer enhancement for a nucleus with arbitrary spin quantum number from n scalar coupled protons for arbitrary preparation times. *J. Magn. Reson.* **44**, 32-40 (1981).
5. Coelho, C., Azaïs, T., Bonhomme-Coury, L., Laurent, G. & Bonhomme, C. Efficiency of the Refocused 31P−29Si MAS-J-INEPT NMR Experiment for the Characterization of Silicophosphate Crystalline Phases and Amorphous Gels. *Inorg. Chem.* **46**, 1379-1387 (2007).
